# Discovery of catalytic-site-fluorescent probes for tracing phosphodiesterase 5 in living cells†

**DOI:** 10.1039/d1ra06247f

**Published:** 2021-10-04

**Authors:** Meiying Qiu, Deyan Wu, Yi-You Huang, Yue Huang, Qian Zhou, Yijing Tian, Lei Guo, Yuqi Gao, Hai-Bin Luo

**Affiliations:** School of Pharmaceutical Sciences, Sun Yat-Sen University Guangzhou 510006 China gaoyuqi@thelilab.cn gaoyq25@mail.sysu.edu.cn luohb77@163.com luohb77@mail.sysu.edu.cn; School of Pharmaceutical Sciences, Hainan University Haikou 570228 China

## Abstract

Small molecule fluorescent probes provide a powerful labelling technology to enhance our understanding of particular proteins. However, the discovery of a proper fluorescent probe for detecting PDE5 is still a challenge due to the highly conservative structure of the catalytic domain in the phosphodiesterase (PDE) families. Herein, we identified probes based on the key amino residues in the ligand binding pocket of PDE5 and catalytic-site-fluorescent probes PCO2001–PCO2003 were well designed and synthesized. Among them, PCO2003 exhibited extraordinary fluorescence properties and the ability to be applied to PDE5 visualization in live cells as well as in pulmonary tissue slices, demonstrating the location and expression level of PDE5 proteins. Overall, the environment-sensitive “turn-on” probe is economical, convenient and rapid for PDE5 imaging, implying that the catalytic-site-fluorescent probe will have a variety of future applications in pathological diagnosis as well as drug screening.

## Abstract 2

To enhance the understanding of PDE5 as the drug target. Herein, we designed catalytic-site-fluorescent probes that can be applied to PDE5 visualization in live cells and tissue slices, implying the potential in diagnosis and drug screening.

## Graphical abstract

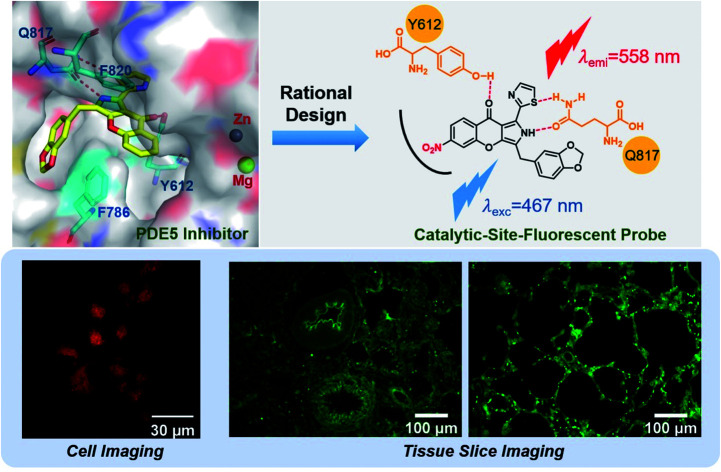

## Introduction

For humans, information visible to the naked eye is always more convictive. It is usually impossible to obtain visual signals of most biomolecules, cells and organisms by direct observation. Currently, opportunities to visualize many physiological processes have been provided by labelling techniques, such as the isotope method, dye staining and fluorescent protein labelling.^1^ More recently, small molecule fluorescent probes have emerged as a powerful labelling technology to enhance our understanding of particular proteins of interest because of their high sensitivity, structural flexibility and simple operation, compared with other types of probes for non-invasive imaging such as positron emission tomography (PET) or nuclear magnetic resonance (NMR).^2^ Comparing with conventional immunofluorescence imaging or fluorescent protein labeling imaging, it's more convenient and superior to use non-invasive fluorescent probes to detect proteins in live cells without the assistance of specific antibodies applied in fixed cells or complicated protein tag techniques.^3^ So far, they have been widely used in protein labelling, bioactive molecule (ROS, H_2_S, NO, DNA, RNA, *etc*)^4–7^ detection and enzyme direct imaging,^8–11^ for dynamic tracing significant biological processes in cells or even live animals.

Phosphodiesterases (PDEs) are a series of vital enzymes that perform unique roles in the signal transduction and duration for many physiopathological processes,^12^ by maintaining the intracellular levels of cyclic nucleotides (cAMP and cGMP) which are involved in cell proliferation and differentiation, cell-cycle regulation, gene expression, inflammation, apoptosis, and metabolism. PDE5 is one of the PDEs family members which specifically catalyses the hydrolysis of cGMP. Currently, several PDE5 inhibitors have been approved for several diseases, such as erectile dysfunction and pulmonary arterial hypertension (PAH).^13,14^ And recently, the role of PDE5 in idiopathic pulmonary fibrosis (IPF) has attracted much attention.^15,16^ It is highly desirable to develop fluorescent probes that can detect and image PDE5 in living biosystems accurately and selectively.

To date, phosphodiesterase activity based probes^17,18^ and cGMP-based probes^19,20^ have been reported. However, the signals of enzymatic fluorescent probes reflect the accumulation of biochemical products and the activity of the total enzymes, rather than the target itself. The discovery of a proper fluorescent probe for detecting the intracellular profile of PDE5 is still a challenging task. The optical probes for tracing and imaging PDE5 were barely reported. Due to the highly conservative structure of catalytic domain in the PDE families, fluorescence labelled cyclic nucleotides may not be specifically recognized by the PDE enzymes, including PDE5. Moreover, many PDEs inhibitors do not have the characteristics of fluorescence, making them impossible to be used as fluorescent probes. Actually, it is still difficult to design a fluorescent probe with a good PDEs inhibitory and specificity based on the structures of PDEs inhibitors.

**Fig. 1:**
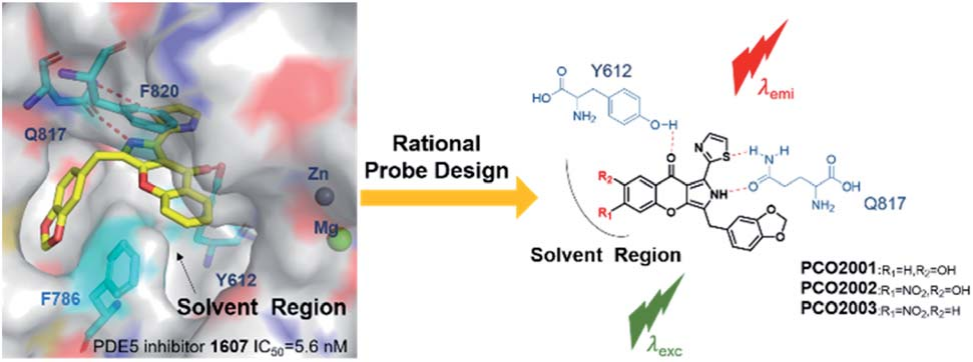
Rational design of catalytic-site-fluorescent probe PCO2001–2003. For LW1607, *R*_1_ = *R*_2_ = H; PCO2001, *R*_1_ = H, *R*_2_ = OH; PCO2002, *R*_1_ = NO_2_, *R*_2_ = OH; PCO2003, *R*_1_ = NO_2_, *R*_2_ = H.

To further understand the real-time distribution and manners of PDE5, especially in pulmonary tissues, we intended to design PDE5-targeting fluorescent probe. In our previous work, we discovered a series of PDE5 inhibitors with chromeno[2,3-*c*]pyrrol-9(2*H*)-one as the skeleton, which exhibited extraordinary inhibition towards PDE5.^21,22^ Surprisingly, LW1607, one of such PDE5 inhibitors, was capable of emitting blue fluorescence under the excitation at 365 nm. However, the capability of LW1607 for fluorescent imaging is still limited by the short-wavelength fluorescent emission and weak fluorescence intensity (Table S1, ESI†). With the help of molecular docking and molecular dynamics (MD) simulations, it is found that the pyrrole scaffold contributes to the excellent binding affinity to PDE5 protein while the chromone scaffold expands to the hydrophobic region where the space is enough for the substituents on the circle (Fig. S2, ESI†). Therefore, it is more suitable to introduce an electron-withdrawing group on the chromone scaffold in order to facilitate π-electron delocalization for intramolecular charge transfer (ICT). With the purpose of preliminarily understanding the structure–activity relationship of these probes, we introduced different substituents to the 6- or 7-position of the chromone moiety to afford three compounds: PCO2001, PCO2002 and PCO2003, respectively. Perhaps we have to compromise on inhibitory activity to endow the compounds with the fluorescence. As shown in Table S1,† the introduction of hydroxyl at position C7 resulted in stronger inhibitions than LW1607, while PDE5 inhibitory activities exhibited weaker by substituting the C6 position with nitro group. It may imply that PDE5 inhibitory activities decreases with the electron density decreases in solvent region (Tables S2 and S3, ESI†). However, the probes become more environment-sensitive and the fluorescent imaging in living cells are better with the electron density decreases in solvent region. Finally, probe PCO2003 was selected for the subsequent experiments because of its excellent “turn-on” effects *in vitro*. It was also applied into detection and visualization of intracellular PDE5 and imaging of rat lung tissue slice, indicating the good ability to monitor PDE5 in a visible manner. This is the first attempt to endow a PDE5 inhibitor with fluorescent properties to establish the fluorescent probe to realize the visual track of the target protein (Fig. 1).

## Results and discussion

### Design and synthesis

These fluorescent probes were well designed and synthesized on the base of the pattern of interaction between LW1607 and the catalytic domain of PDE5. The detail synthesis procedure was explained in the ESI (Fig. S1†). All these probes could be predicted to have good electro-static interactions in allusion to amino acid residues of the catalytic pocket in PDE5 protein, contributing to the strong receptor-ligand binding patterns (Fig. S2, ESI†). These typical interactions include H-bond interactions for Y612 and Q817, and π–π interactions for F820 and F786. The pyrrole chromone scaffold matched well with the catalytic pocket in PDE5 protein contributing to a desired receptor–ligand binding pattern. The rigid and π-conjugated tricyclic conjugate scaffold is essential for the optical property.

### Spectroscopic properties of the probes

**Fig. 2:**
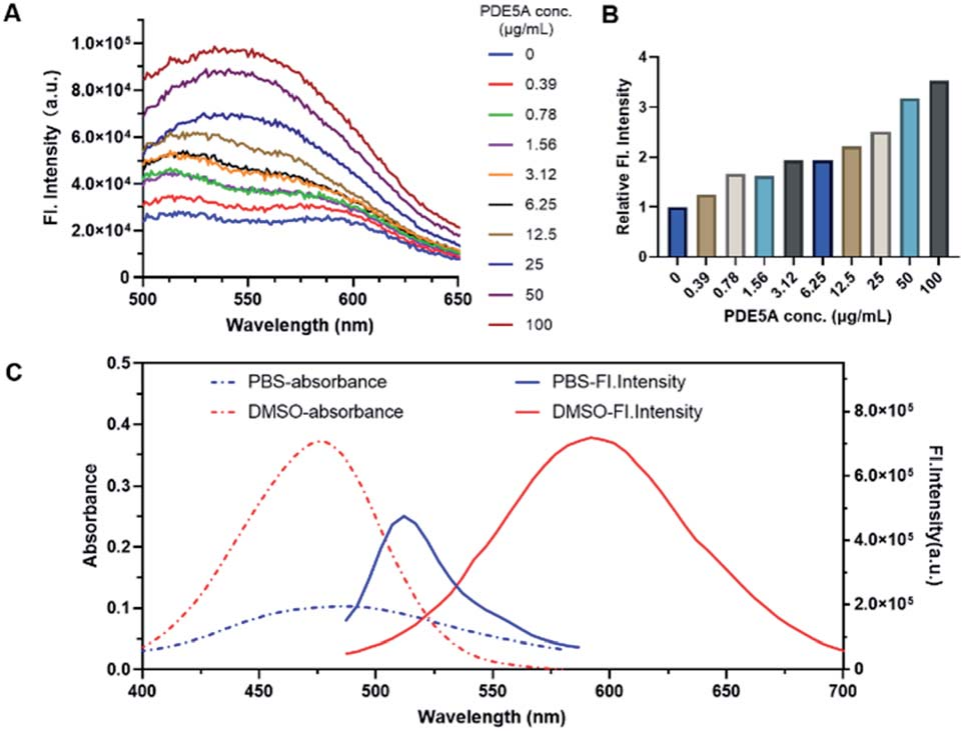
(A) Fluorescent spectra of probe PCO2003 (10 μM) in PBS (1×, pH 7.4) when incubated with different concentration of recombinant PDE5A protein from 0 to 100 μg mL^−1^. (B) The relative fluorescence intensity of probe PCO2003 (10 μM) in PBS when incubated with different concentration of recombinant PDE5 protein from 0 to 100 μg mL^−1^. The fluorescence intensity was obtained at 558 nm. (C) Absorption and fluorescence spectra of PCO2003 in PBS and DMSO.

All the probes performed the good environmentally sensitivity towards different polarity solvents (Table S1, Fig. S3 and S4, ESI†). The results suggested the potency of the compounds as a type of environment-sensitive turn-on probes. Exhibiting the excellent optical property among the three probes, PCO2003 was selected for the subsequent experiments. When the probe PCO2003 was incubated with recombinant PDE5 protein in PBS (1×, pH 7.4), the fluorescence intensity gradually increased with the augmentation of the loaded amount of PDE5 protein, and reached more than 3-fold at the concentration of 0.1 mg mL^−1^ over PBS (Fig. 2), indicating an excellent binding pattern between this probe and PDE5 as well as the potential of fluorescent imaging in cells and even tissues.

### Visualization of PDE5 in live cells with PCO2003

**Fig. 3:**
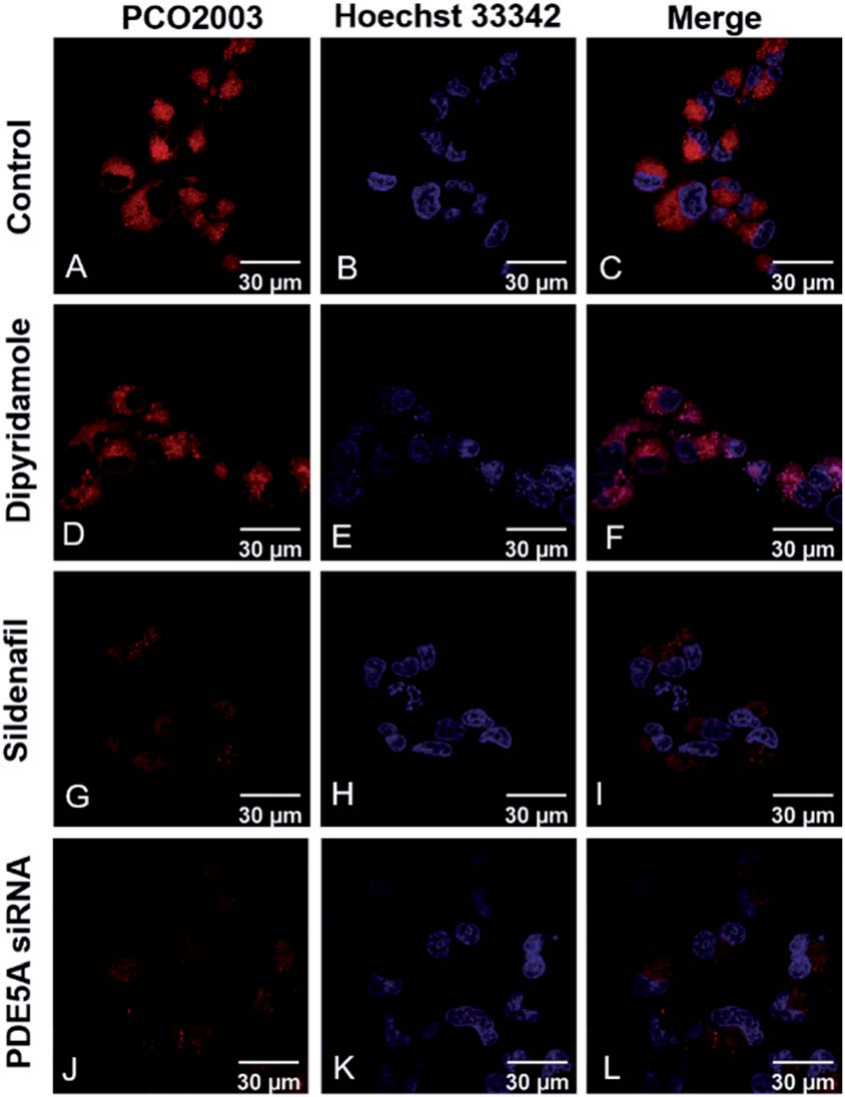
Fluorescent images of live LX-2 cells stained with PCO2003 (10 μM) (A, D, G, J) and Hoechst 33342 (B, E, H, K). Fluorescent images of live LX-2 cells (A–C) as a control group; (D–F) LX-2 cells coincubated simultaneously with the PDE5A inhibitor dipyridamole (25 μM); (G–I) LX-2 cells coincubated simultaneously with the PDE5A inhibitor sildenafil (25 μM); (J–L) transiently transfected LX-2 cells with siRNA for the corresponding PDE5A knockdown group. The scale bar is 30 μm. For each sample, approximately 50 cells were measured.

Fluorescent imaging assays were conducted on LX-2, A549 and HeLa cells by laser-scanning confocal microscopy (LSCM). The human hepatic stellate cell line LX-2 was purchased from Procell Life Science Technology. The human lung carcinoma cell line A549 was purchased from American Type Culture Collection (ATCC). The human cervical carcinoma cell line HeLa was purchased form National Collection of Authenticated Cell Cultures. All the probes exhibited low cytotoxicity, indicating the good biological compatibility for bio-imaging assays (Fig. S5, ESI†). Except PCO2001, the other probes were capable to penetrate the membrane of live LX-2 cell and track PDE5 in a visible manner (Fig. S6 and S7, ESI†). In different cells, PCO2003 showed augment fluorescence intensity in line with the increased intracellular expression levels of PDE5 (Fig. S8†). We also explored the interference of PDE5 inhibitors towards PCO2003 (Fig. 3) and found that the fluorescent staining in live LX-2 cells was significantly influenced by sildenafil (PDE5, IC_50_ = 2.5 nM) but not inhibited competitively by dipyridamole (PDE5, IC_50_ = 211 nM). Moreover, when LX-2 cells were transiently transfected with PDE5 siRNA to knock down the target protein before fluorescent imaging, the fluorescent signals of PCO2003 declined obviously compared to the control group (Fig. 3 and S9, ESI†). The fluorescent imaging assays indicated the specificity of PCO2003 towards PDE5 in live cells.

### Tissue slice imaging with PCO2003

**Fig. 4:**
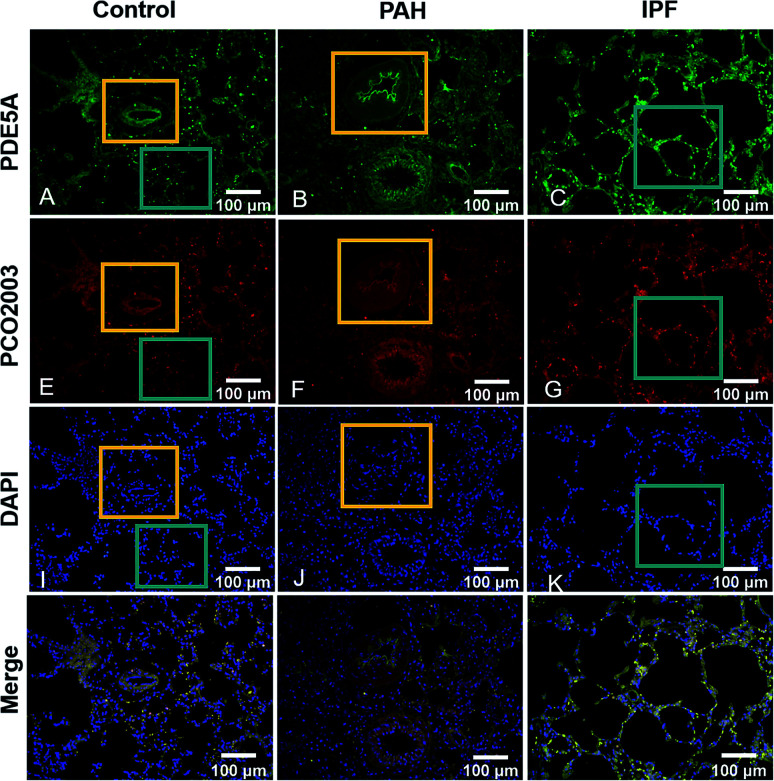
Immune fluorescence images of pulmonary tissue stained with PDE5A antibody (A–C), PCO2003 (10 μM) (D–F) and DAPI (G–I), as well as the merge images (J–L). The fluorescent images refer to normal pulmonary tissues (A, D, G, J), MCT-induced pulmonary arterial hypertension tissues (B, E, H, K) and BLM-induced idiopathic pulmonary fibrosis tissues (C, F, I, L). In the orange boxes, the pulmonary artery was marked, while the pulmonary alveoli were marked in the cyan boxes.

As is reported that some lung-related diseases such as pulmonary arterial hypertension (PAH) and idiopathic pulmonary fibrosis (IPF) are associated with the up-regulation of PDE5,^15,23,24^ indicating the potency of PDE5 as the novel targets for the treatment of the diseases. Subsequently, we further explored the capability of PCO2003 staining for rat pulmonary tissue slices. Apparently, the fluorescence signals of PCO2003 as well as PDE5 fluorescent anti-antibody significantly increased in monocrotaline (MCT)-induced PAH tissues slices and bleomycin (BLM)-induced IPF tissue slices, compared with those of the control group, demonstrating the selectivity of PCO2003 towards PDE5 in tissues. The direct fluorescent image of PDE5 stained by PCO2003 could be applied to reflect the levels of lung damage and fibrosis in IPF (displayed in the cyan boxes) as well as the thickened pulmonary arteries (displayed in the orange boxes) in PAH, which gave morphological evidence to support PDE5 as a novel efficient target for some lung-related diseases (Fig. 4). The tissue sections imaging results also indicated that the fluorescent probe PCO2003 could be an efficient substitute for the antibodies in immune fluorescent imaging, for its extraordinary specificity and simple operation.

## Conclusions

In summary, based on the insights into the ligand binding pocket of PDE5, three novel small-molecule fluorescent probes for PDE5 visualization were designed and synthesized. Among them, PCO2003 exhibited reasonable fluorescent properties and high feasibility in detection and visualization of PDE5 protein. The results of bioactivity evaluation indicated the capability of the probe to fluorescent imaging in live cells and tissue slices. Moreover, the “turn-on” PDE5-specific fluorescent probe PCO2003 is more convenient, rapid and economical as a visualization tool for PDE5, compared to the immunofluorescence or fluorescent protein-based techniques. These findings implying that the catalytic-site-fluorescent probe will have a variety of future applications in pathological and physiological studies as well as screening of other PDE5 inhibitors.

## Conflicts of interest

The authors declare no competing financial interest.

## Supplementary Material

RA-011-D1RA06247F-s001
