# Supplementary material for: Discovery of catalytic-site-fluorescent probes for tracing phosphodiesterase 5 in living cells
Source: RSC Adv. 2021 Oct 4;11(51):31967–71. doi: 10.1039/d1ra06247f (PMC9041563; doi:10.1039/d1ra06247f)
Supplement: RA-011-D1RA06247F-s001 [file RA-011-D1RA06247F-s001.pdf]

Electronic Supplementary Material (ESI) for RSC Advances.
This journal is © The Royal Society of Chemistry 2021

Discovery of Catalytic-Site-Fluorescent Probes for Tracing Phosphodiesterase 5 in Living Cells

Meiying Qiu[a, #], Deyan Wu[a,b,#], Yiyou Huang[a,b], Yue Huang[a], Qian Zhou[a], Yijing Tian[a], Lei Guo[a], Yuqi Gao[a,*], Hai-Bin Luo[a,b,*]

[a.] School of Pharmaceutical Sciences, Sun Yat-Sen University, Guangzhou 510006, China.

[b.] School of Pharmaceutical Sciences, Hainan University, Haikou 570228, China.

[#.] These authors equally contributed to this work.

E-mail: gaoyuqi@thelilab.cn or gaoyq25@mail.sysu.edu.cn (Y. Gao);
luohb77@163.com or luohb77@mail.sysu.edu.cn (H.-B. Luo).

Contents

1. Supplementary spectra and graphs and other data 2
2. Materials and instruments....................6
3. Synthesis of probes **PCO2001-2003**....................7
4. Experiment section 10
4.1 Protein expression and purification....................10
4.2 Fluorescent excitation and emission spectra of **PCO2001-2003** 10
4.3 The fluorescence quantum yields measurements....................10
4.4 Fluorescence properties of **PCO2003** with PDE5A recombinant protein 11
4.5 Cell culture....................11
4.6 Cytotoxicity evaluation 11
4.7 Cell fluorescent imaging....................11
4.8 Cell immunofluorescence imaging....................12
4.9 Cell transiently transfected with PDE5A siRNA....................12
4.10 Real-time quantitative RT-PCR assays 12
4.11 Western Blot assays....................13
4.12 Tissue sections immunofluorescence imaging....................13
5. Appendix....................14
6. References 24

1.Supplementary spectra and graphs and other data

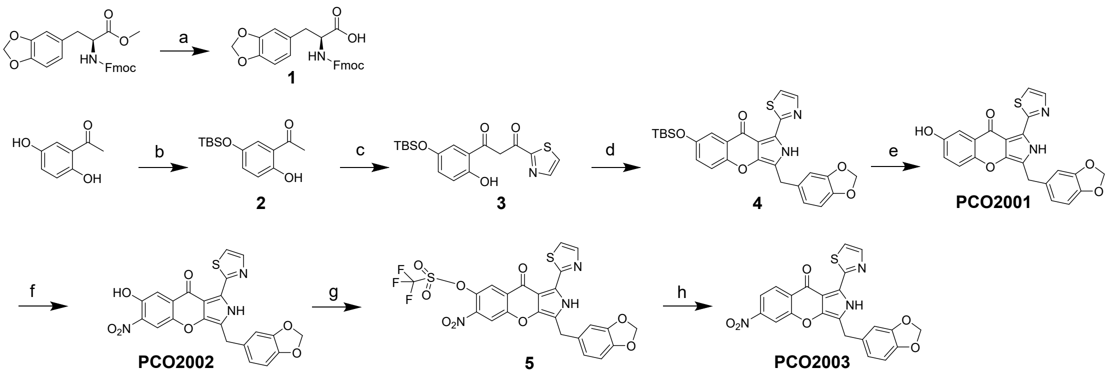

Fig. S1 Synthesis details of PDE5A catalytic-site targeting fluorescence probes **PCO2001-2003**. Reagents and conditions:(a) KOH, $CaCl_2$, THF, H_2O, RT; (b)TBSCl, imidazole, THF, 4 h; (c) ethyl thiazole-2-carboxylate, NaH, toluene, RT, 30 min, 65 °C, 2 h; (d) **1**, *N*-(3-dimethylaminopropyl)-N'-ethylcarbodiimide hydrochloride (EDCI), 4-dimethylaminopyridine (4-DMAP), pyridine, RT, 3 h, 55°C, overnight; (e) Tetrabutylammonium fluoride (TBAF), THF, 1 h; (f) 30% H_2SO_4, 20% $NaNO_2$, pyridine,3 h; (g)CH_2Cl_2, Trifluoromethanesulfonic anhydride, pyridine, RT, 15 min. (h) $Pd(OAc)_2$, Triethylamine, diphenyl-1-pyrenylphosphine (dppp), Formic acid, 60°C, 2 h.

Table S1. Fluorescent properties of probe **PCO2001-2003**.

Compound	λ_{exc} (nm)	λ_{emi} (nm)	Quantum yield in PBS
**PCO2001**	467	506	3.67%
**PCO2002**	468	576	1.26%
**PCO2003**	467	558	0.70%
**LW1607**	428	508	1.82%

Table S2. IC_{50} of **PCO2001-2003**.

	LW1607	PCO2001	PCO2002	PCO2003
IC_{50} (nM)	5.6 ± 0.3 nM	1.1 ± 0.1 nM	7.1 ± 1.2 nM	65.4±5.0 nM

Table S3. Selectivity assessment of **PCO2003**.

	IC_{50}	Selectivity
PDE1	>10 µM	>100
PDE2	>10 µM	>100
PDE4	2.2±0.1µM	30
PDE5	65.4±5.0 nM	
PDE7	>10 µM	>100
PDE8	>10 µM	>100
PDE9	>10 µM	>100
PDE10	700 nM	10

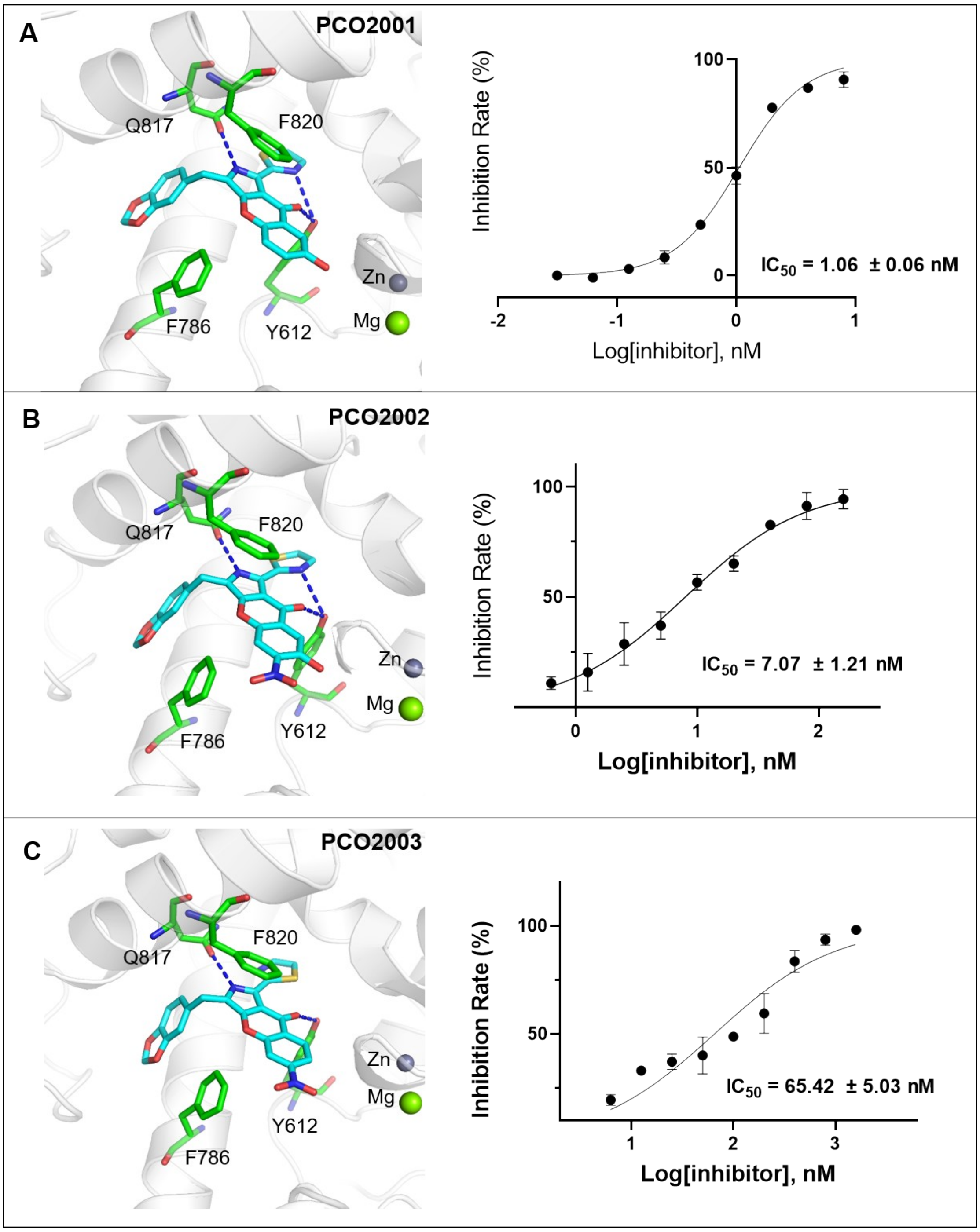

Fig. S2 The diagram of molecular docking between probes and PDE5A protein and the inhibitory activity of the probes towards PDE5A. Values of PDE5A inhibitory rate are presented as the means ± SD (n = 2) with as a rolipram control (IC_{50} = 800 nM).

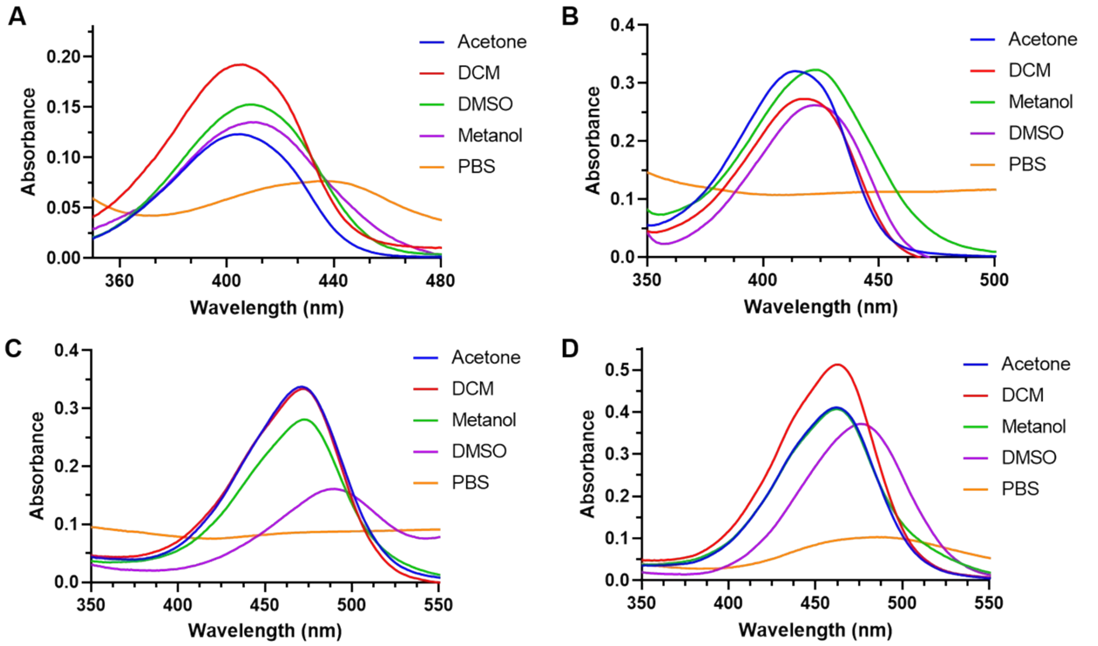

Fig. S3. Absorption spectra in different solvents: (A) **LW1607**, (B) **PCO2001**, (C) **PCO2002**, (D) **PCO2003**.

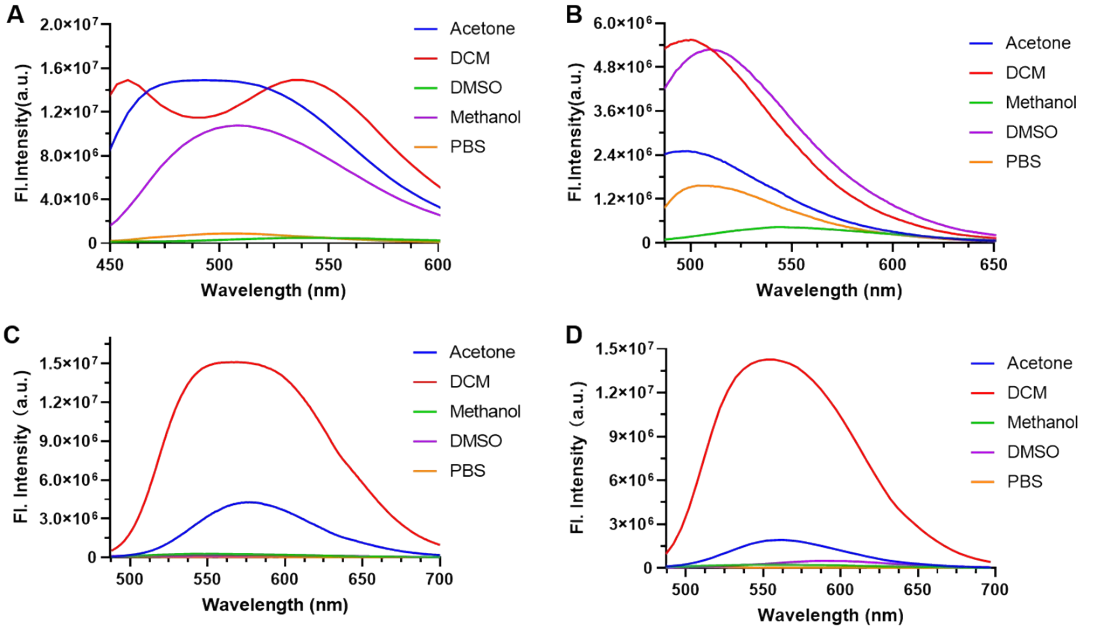

Fig. S4. Fluorescence spectra in different solvents: (A) **LW1607**, (B) **PCO2001**, (C) **PCO2002**, (D) **PCO2003**.

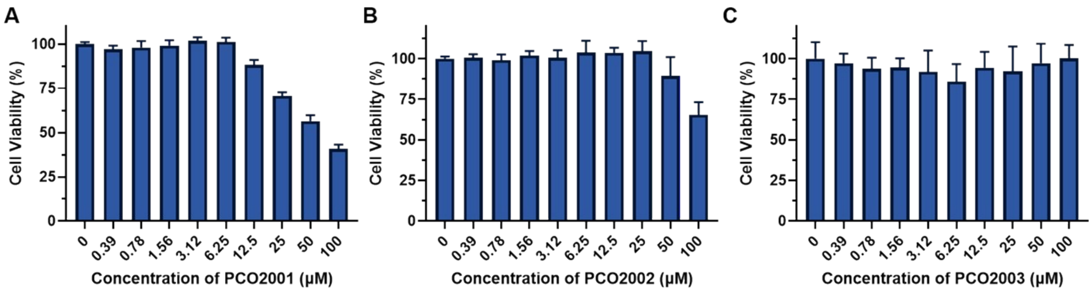

Figure S5. Cytotoxicity assay of (A) **PCO2001**, (B) **PCO2002** and (C) **PCO2003** in LX-2 cells after a 24 h incubation

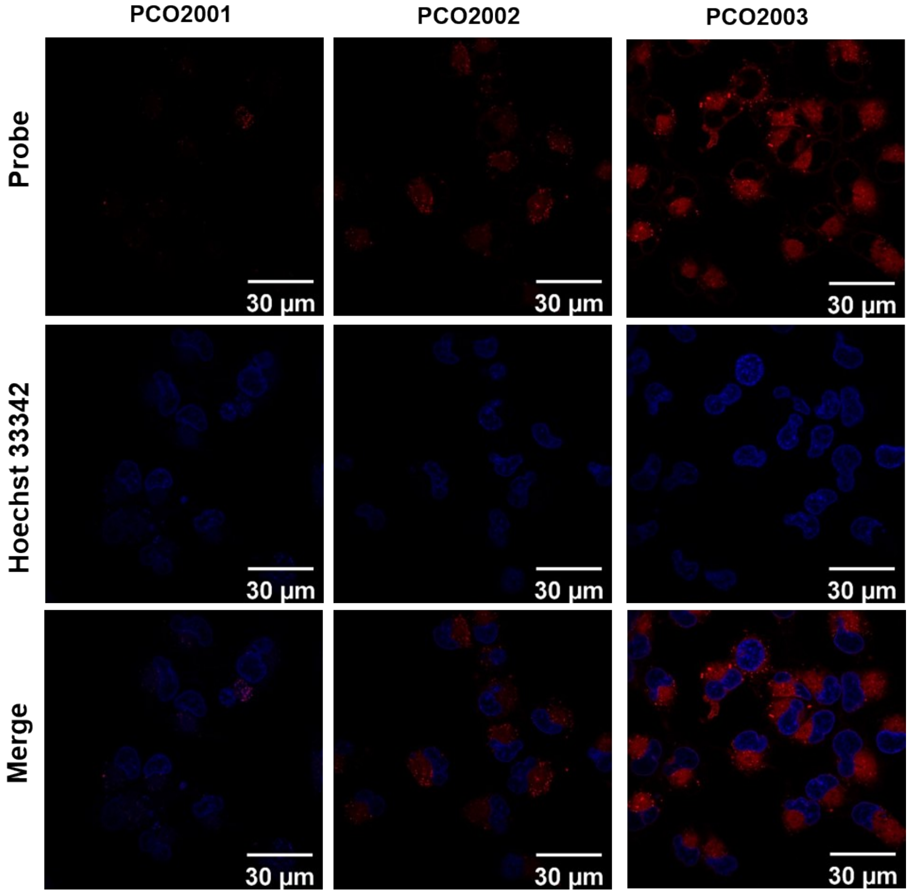

Figure S6. Fluorescent cell imaging co-stained with probes and Hoechst 33342 in live LX-2 cells.

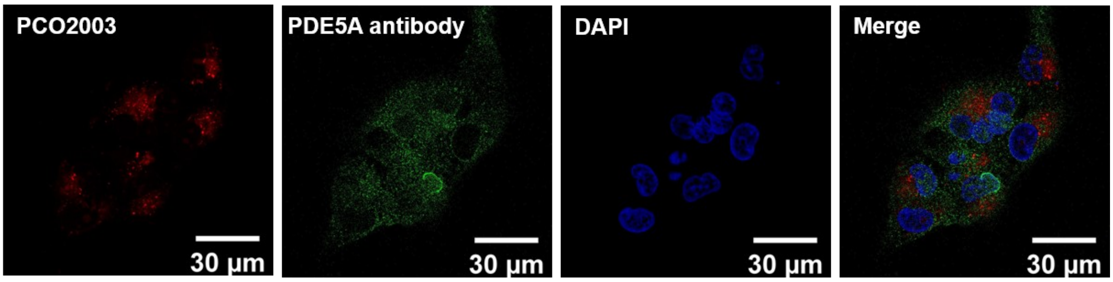

Figure S7. Immune fluorescence images in fixed LX-2 cells.

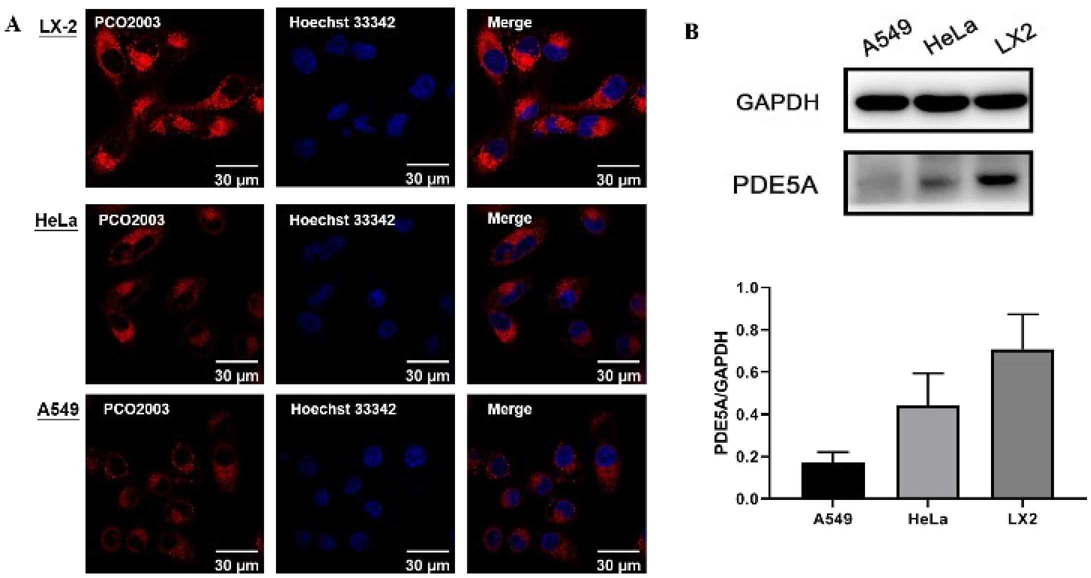

Figure S8. (A) Fluorescent cell imaging co-stained with **PCO2003**(10 μM) and Hoechst 33342 by a confocal fluorescence microscope at the same condition in LX-2 cells, HeLa cells, A549 cells. (B) The PDE5A expression in different cells analyzed with Western Blot method.

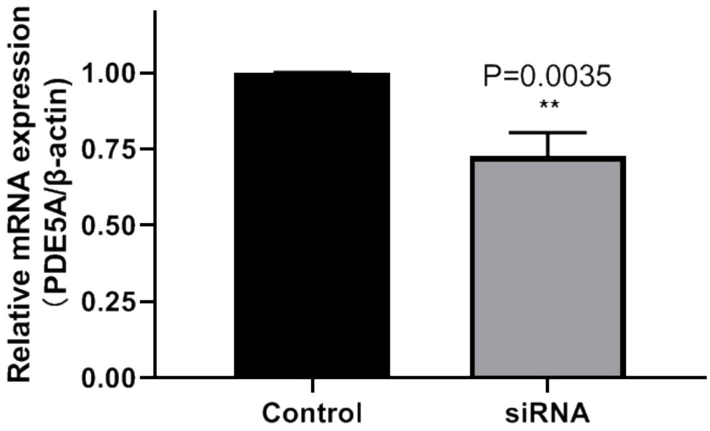

Figure S9. Interference effect of PDE5A siRNA

2. Materials and instruments

All reagents and solvents are commercially available and used as received unless otherwise noted. Water used for the fluorescence and biological studies was doubly distilled and further purified with a Mill-Q filtration system. Fluorescence spectra were obtained under the fluorescence spectrograph HORIBA Fluoromax-4. The CCK-8 cytotoxicity assay was conducted on a microplatereader (POLARstar, Omega). Fluorescence imaging was performed by using the fluorescence microscopy (DMI-3000B, OLYMPUS) and the confocal fluorescence microscopy (FV-3000, OLYMPUS).

Quantitative Real-time PCR (qRT-PCR) assays were performed by using qRT-PCR system (LightCycler480II, Roche).

3. Synthesis of probes PCO2001-2003

(S)-2-((((9H-fluoren-9-yl)methoxy)carbonyl)amino)-3-(benzo[d][1,3]dioxol-5-yl)propanoic acid (1)[1].

To a solution of $CaCl_2$(2.2 g, 20 mmol), KOH(448 mg,8 mmol) in water (20 mL) was added methyl (S)-2-((((9H-fluoren-9-yl)methoxy)carbonyl)amino)-3-(benzo[d][1,3]-dioxol-5-yl)propanoate(1.78 g, 4 mmol) in THF (8 mL) slowly at 0 °C. Then the reaction mixture was allowed to warm to room temperature and stirred overnight. Upon completion of the reaction, it was acidified by the addition of 2 mol/L aqueous HCl. The resulting solution was extracted with ethyl acetate and washed with water, brine and dried over anhydrous Na_2SO_4 and was concentrated. Then, it was added the solution of n-hexane: ethyl acetate=20:1 and stirred for 4 h. The mixture was filtered to afford the desired **1** as white solid(1.7 g, 95%). ^{1}H-NMR (500 MHz, $CDCl_3$): δ 7.77 (d, *J* = 7.5 Hz, 2H), 7.56 (t, *J* = 7.9 Hz, 2H), 7.40 (t, *J* = 7.4 Hz, 2H), 7.31 (dt, *J* = 11.7, 5.9 Hz, 2H), 6.72 (d, J = 7.8 Hz, 1H), 6.64 (s, 1H), 6.58 (d, *J* = 7.7 Hz, 1H), 5.90 (s, 2H), 5.26 (d, *J* = 8.1 Hz, 1H), 4.66 (dd, *J* = 13.2, 5.7 Hz, 1H), 4.46 (dd, *J* = 10.6, 7.3 Hz, 1H), 4.37 (dd, *J* = 10.4, 7.1 Hz, 1H), 4.21 (t, *J* = 6.9 Hz, 1H), 3.11 (dd, *J* = 14.0, 5.3 Hz, 1H), 3.05 (dd, *J* = 14.1, 5.8 Hz, 1H).

1-(5-((tert-butyldimethylsilyl)oxy)-2-hydroxyphenyl)-3-(thiazol-2-yl)propane-1,3-dione (2)[2].

TBSCl (729 mg, 4.8 mmol) was slowly added into a solution of 1-(2,5-dihydroxyphenyl)ethan-1-one(669 mg, 4.4 mmol) and imidazole (449 mg, 6.6 mmol) in THF (25 mL) at 0 °C. Then the reaction mixture was allowed to warm to room temperature and stirred overnight. Upon completion of the reaction, the reaction mixture was extracted with ethyl acetate. The solution was washed with water, brine and dried over anhydrous Na_2SO_4. The organic extract was concentrated under reduced pressure and the crude product was purified by silica gel flash column chromatography (PE:EA, 20:1) to afford the desired **2** as light yellow oil(1.1 g, 93% yield). ^{1}H-NMR (500 MHz, $CDCl_3$): δ 11.83 (s, 1H), 7.15 (d, *J* = 2.9 Hz, 1H), 7.01 (dd, *J* = 8.9, 2.9 Hz, 1H), 6.86 (d, *J* = 8.9 Hz, 1H), 2.59 (s, 3H), 0.99 (s, 9H), 0.19 (s, 6H).

1-(5-((tert-butyldimethylsilyl)oxy)-2-hydroxyphenyl)-3-(thiazol-2-yl)propane-1,3-dione (3)[1].

To a solution of 1-(5-((tert-butyldimethylsilyl)oxy)-2-hydroxyphenyl)ethan-1-one (1.1g, 4.3 mmol) in dry toluene (200 mL) was added NaH (60% in mineral oil, 860 mg, 21.5 mmol) slowly at 0 °C**.** Then the reaction mixture was stirred at room temperature for 30 min and the solution of ethyl thiazole-2-carboxylate(810 mg, 4.7 mmol) in dry toluene(22 mL) was dropwise added and the mixture was stirred at 65 °C for 2 h. After

the solution cooled to room temperature, it was poured into a mixture of ice and water and acidified by the addition of 2 mol/L aqueous HCl. The resulting solution was extracted with ethyl acetate. The combined organic phase was washed with water, brine and dried over anhydrous Na_2SO_4 and was concentrated. Then the crude product was purified by silica gel flash column chromatography (PE: EA, 6:1 to 2:1) to give the product **3** as a yellow solid (1.3 g, 80% yield). ^{1}H-NMR (400 MHz, $CDCl_3$): δ 11.49 (s, 1H), 8.07 (d, *J* = 3.1 Hz, 1H), 7.69 (d, *J* = 3.1 Hz, 1H), 7.30 – 7.29 (m, 1H), 7.06 (dd, *J* = 8.9, 2.9 Hz, 1H), 6.93 – 6.90 (m, 1H), 1.03 (s, 9H), 0.24 (d, *J* = 3.0 Hz, 6H).

3-(benzo[d][1,3]dioxol-5-ylmethyl)-7-((tert-butyldimethylsilyl)oxy)-1-(thiazol-2-yl)chromeno[2,3-c]pyrrol-9(2H)-one (4)[1].

To a solution of 1-(5-((tert-butyldimethylsilyl)oxy)-2-hydroxyphenyl)-3-(thiazol-2-yl)propane-1,3-dione(1.3 g,3.5 mmol), methyl(S)-2-((((9H-fluoren-9-yl)methoxy)-carbonyl)amino)-3-(benzo[d][1,3]dioxol-5-yl)propanoate (1.7 g, 3.9 mmol), 4-dimethylpyridine (215 mg, 1.8 mmol) in pyridine (20 mL) was added EDCI(2.0 g, 10.5 mmol), respectively. The mixture was stirred at room temperature for 3 h until the start material disappeared as monitored by TLC. The reaction mixture was heated to 55°C overnight. After the reaction mixture was evaporated under vacuum, the residue was extracted with ethyl acetate and washed with water to remove the remaining pyridine. The solution was evaporated and purified by silica gel flash column chromatography (PE: EA, 5:1) to afford **4** as orange solid (825 mg, 45% yield). ^{1}H-NMR (400 MHz, CDCl3): δ 7.77 (d, *J* = 3.0 Hz, 1H), 7.73 (d, *J* = 3.2 Hz, 1H), 7.35 (d, *J* = 3.2 Hz, 1H), 7.29 (d, *J* = 8.9 Hz, 1H), 7.16 (dd, *J* = 8.9, 3.0 Hz, 1H), 6.56 (d, *J* = 7.9 Hz, 1H), 6.45 (dd, *J* = 19.4, 4.7 Hz, 2H), 5.73 (s, 2H), 3.98 (s, 2H), 1.02 (s, 9H), 0.25 (s, 6H).

3-(benzo[d][1,3]dioxol-5-ylmethyl)-7-hydroxy-1-(thiazol-2-yl)chromeno[2,3-c]pyrrol-9(2H)-one (PCO2001)[3].

Tetrabutylammonium fluoride (1.5 mL, 1 mol/L in THF, 1.5 mmol) was added into a solution of 3-(benzo[d][1,3]dioxol-5-ylmethyl)-7-((tert-butyldimethylsilyl)oxy)-1-(thiazol-2-yl)chromeno[2,3-c]pyrrol-9(2H)-one (825 mg, 1.5 mmol) in THF(8 mL) at room temperature and stirred for 1 h until the start material disappeared as monitored by TLC. Then the solution was extracted with ethyl acetate and washed with water, brine and dried over anhydrous Na_2SO_4. Then the crude product was purified by silica gel flash column chromatography (PE: EA, 1:1) to give the product **PCO2001** as yellow solid (616 mg, 95% yield). ^{1}H-NMR (400 MHz, DMSO): δ 13.00 (s, 1H), 9.76 (s, 1H), 7.91 (d, *J* = 3.2 Hz, 1H), 7.72 (d, *J* = 3.2 Hz, 1H), 7.49 (d, *J* = 2.7 Hz, 1H), 7.40 (d, *J* = 9.0 Hz, 1H), 7.19 (dd, *J* = 9.0, 3.0 Hz, 1H), 6.93 (s, 1H), 6.82 (t, *J* = 7.1 Hz, 2H), 5.94 (s, 2H), 4.09 (s, 2H). ^{13}C-NMR (126 MHz, DMSO): δ 174.69, 157.45, 153.51, 150.30, 147.72, 146.08, 142.76, 141.93, 133.59, 123.56, 122.72, 121.55, 120.78, 119.90, 119.34, 116.10, 109.75, 109.20, 108.91, 108.70, 101.23, 29.40. HRMS (ESI-TOF) m/z $[M+H]^+$ calcd. for $C_{22}H_{14}N_2O_5S$ 419.0696, found 419.0694.

3-(benzo[d][1,3]dioxol-5-ylmethyl)-7-hydroxy-6-nitro-1-(thiazol-2-yl)chromeno[2,3-c]pyrrol-9(2H)-one (PCO2002)[4].

At 0 °C, an aqueous solution of $NaNO_2$ (20%, w/v, 5.1 mL) was added to the solution of 3-(benzo[d][1,3]dioxol-5-ylmethyl)-7-hydroxy-1-(thiazol-2-yl)chromeno[2,3-c]pyrrol-9(2H)-one(616 mg, 1.5 mmol) in dry pyridine (15 mL). Then, 30% (v/v) aqueous H_2SO_4 (0.99 mL) was added dropwise. After stirring for 3h at 0°C, the solution was poured into water full of plenty of ice. Then the precipitate was collected and pyridine was removed by filtration. Then the resulting precipitate was purified by flash chromatography (PE: EA, 2:1) to afford **PCO2002** as red solid (44 mg, 6.5% yield). ^{1}H-NMR (400 MHz, Acetone): δ 8.61 (s, 1H), 7.65 (d, *J* = 3.0 Hz, 1H), 7.45 (d, *J* = 9.0 Hz, 1H), 7.30 (dd, *J* = 9.0, 3.1 Hz, 1H), 6.92 (s, 1H), 6.87 (d, *J* = 8.0 Hz, 1H), 6.76 (d, *J* = 8.0 Hz, 1H), 5.93 (s, 2H), 4.27 (s, 2H). ^{13}C-NMR (126 MHz, DMSO) δ 174.80, 161.87, 153.98, 150.32, 147.78, 146.93, 146.25, 145.41, 143.50, 132.56, 124.16, 122.58, 121.77, 121.71, 119.60, 117.88, 111.44, 109.38, 109.25, 108.75, 101.29, 29.61. HRMS (ESI-TOF) m/z $[M+H]^+$ calcd. for $C_{22}H_{13}N_3O_7S$ 464.0547, found 464.0553.

3-(benzo[d][1,3]dioxol-5-ylmethyl)-6-nitro-9-oxo-1-(thiazol-2-yl)-2,9-dihydro-chromeno[2,3-c]pyrrol-7-yl trifluoromethanesulfonate (5)[5].

To a solution of 3-(benzo[d][1,3]dioxol-5-ylmethyl)-7-hydroxy-6-nitro-1-(thiazol-2-yl)chromeno[2,3-c]pyrrol-9(2H)-one(44 mg,0.095 mmol), pyridine (32 µL, 0.5 mmol) in CH_2Cl_2 (4 mL) was added Tf_2O (84 µL, 0.5 mmol) at 0 °C and then allowed to warm to RT a and stirred for 15 min. The reaction was extracted with ethyl acetate. The combined organic phase was concentrated and dried over anhydrous Na_2SO_4. The crude was purified by silica chromatography (PE: EA, 2:1) to get desired product **5** (54 mg, 95% yield) as red solid. ^{1}H-NMR (400 MHz, $CDCl_3$): δ 11.49 (s, 1H), 8.07 (d, *J* = 3.1 Hz, 1H), 7.69 (d, *J* = 3.1 Hz, 1H), 7.30 – 7.29 (m, 1H), 7.06 (dd, *J* = 8.9, 2.9 Hz, 1H), 6.93 – 6.90 (m, 1H), 1.03 (s, 9H), 0.24 (d, *J* = 3.0 Hz, 6H). HRMS (ESI-TOF) m/z $[M-H]^-$ calcd. for $C_{23}H_{12}N_3O_9F_3S_2$ 593.9894, found 593.9875.

3-(benzo[d][1,3]dioxol-5-ylmethyl)-6-nitro-1-(thiazol-2-yl)chromeno[2,3-c]pyrrol-9(2H)-one (PCO2003) .

3-(benzo[d][1,3]dioxol-5-ylmethyl)-6-nitro-9-oxo-1-(thiazol-2-yl)-2,9-dihydro-chromeno[2,3-c]pyrrol-7-yl trifluoromethanesulfonate (54 mg, 0.09 mmol), $Pd(OAc)_2$ (6.1 mg, 0.027 mmol), Triethylamine（250 µL,1.8 mmol）and dppp (19 mg, 0.045 mmol) were dissolved in anhydrous DMF (3 mL) under nitrogen atmosphere. Next, Formic acid (6.1 µL, 0.162 mmol) was added to the mixture and stirred at 60 °C for 2 h. The mixture was extracted with ethyl acetate and washed with brine. The combined organic phase was concentrated and dried over anhydrous Na_2SO_4. The crude was purified by silica chromatography (PE: EA, 2:1) to give compound **PCO2003** (12 mg, 31% yeild) as red solid. ^{1}H-NMR (400 MHz, DMSO): δ 13.71 (s, 1H), 8.90 (s, 1H), 8.23 (s, 1H), 7.82 (s, 1H), 7.62 (s, 1H), 7.45 (s, 1H), 6.90 (d, *J* = 50.7 Hz, 3H), 5.96 (s, 2H), 4.16 (s, 2H). ^{13}C-NMR (101 MHz, DMSO): δ 174.90, 161.78, 156.76, 147.82, 147.18, 146.29, 145.43, 143.20, 135.47, 132.52, 126.52, 124.30, 122.01, 121.90, 121.76, 118.54, 118.32, 111.72, 109.29, 108.79, 101.31, 29.60. HRMS (ESI-TOF) m/z $[M+H]^+$ calcd. for

$C_{22}H_{13}N_3O_6S$ 448.0598, found 448.0606.

4. Experiment section

4.1 Protein expression and purification

The expression and purification of PDE5A were carried out similarly to previously published protocols[6]. Briefly, the catalytic domain coding (535-860) of PDE5A was cloned to vector pET-15b and then the cDNA was transferred to E. coli strain BL21 (CodonPlus, Stratagene) for overexpression. When the cell carrying the plasmid was grown in LB medium at 37°C until OD600=0.6-0.8, 0.1mM isopropyl b-D-thiogalactopyranoside (IPTG) was added to induce PDE5A expression for further 20 h growth at 15 °C. PDE5A protein was purified through Ni-NTA column (=2.5 cm, 15 Ml QIAGEN agarose beads), Q-column (2.5 × 8 cm, GE Healthcare) and Superdex 200 column (2.5 × 45 cm, GE Healthcare). The catalytic domains of PDE1B (10-487), PDE2A (580-919), PDE3A (679-1087), PDE4D (86-413), PDE9A (181-506), and PDE10A (449-770) were purified by the similar protocol. PDE8A (480-820) was expressed and purified following the protocol published previously.

4.2 Fluorescent excitation and emission spectra of PCO2001-2003

DMSO stock of probe compounds (20 mM) was diluted to 20 µM with various solvents, namely, acetone, DCM, methanol, DMSO and PBS. Then, the spectra were scanned with UV/VIS spectrophotometer and fluorescence spectrophotometer.

4.3 **The fluorescence quantum yields measurements**

To prepare test samples, DMSO stock of probe PCO2001-2003(20 mM) was diluted with PBS buffer (1×, pH 7.4) and fluorescein sodium (Φ470 =0.92) with 0.1 M NaOH until OD $_{max}$=0.03-0.05 measured by UV/VIS spectrophotometer. Then, the spectra were scanned with fluorescence spectrophotometer. The fluorescence quantum yield (QY) can be calculated from equation:

$$\phi_x = \phi_i \cdot \frac{A_i F_x}{A_x F_i}\left(\frac{n_x}{n_i}\right)^2$$

Where ϕ_x and ϕ_i are the fluorescence QY of the sample and the standard, F_x and F_i are the integrated intensities (areas) of sample and standard spectra, respectively (in units of photons); A_x and A_i are the absorption factor, the refractive indices of the

sample and reference solution are n_x and n_i, respectively.

4.4 **Fluorescence properties of PCO2003 with PDE5A recombinant protein**

PDE5A recombinant protein was prepared in our group following the protocol published previously. PDE5A protein were diluted to different concentration from 0 to 0.2 mg/mL and incubated with probe **PCO2003** (20 μM) in PBS buffer (1×, pH 7.4). After blending the two samples in a quartz cell, the fluorescent spectra were scanned with fluorescence spectrophotometer at 467 nm.

4.5 Cell culture

LX-2 cells, A549 cells and HeLa cells were cultured in DMEM medium with 10% FBS(v/v). All the cells were cultivated in an incubator under 5% CO_2 at 37 °C.

All the medium used for cell culture contained 1% penicillin-streptomycin (v/v) unless otherwise noted.

4.6 Cytotoxicity evaluation

Cell viability assay was performed to evaluate the cytotoxicity of PCO2001-2003 by CCK8 method. Cells were planted into a 96-well cell culture plate at approximate 6000 cell/well in 100 μL medium. The cells were incubated for 12 h at 37°C under 5% CO_2 in an incubator. Then, a solution of PCO2001-2003 (100 μL/well) at concentrations of 0, 0.39, 0.78, 1.56, 3.14, 6.25, 12.5, 25, 50, 100 μM in DMEM medium was added to the wells, respectively. After 24 h incubation, cell viability was measured by CCK-8 method.

4.7 Cell fluorescent imaging

Cells were seeded into a confocal dish at 1 × 10^5/ per dish in 1 mL medium and incubated for 24 h. After the medium was removed, the cells were carefully washed with 37 °C PBS (1×, pH 7.4), and Subsequently the cells were incubated with **PCO2003**(10 μM) and Hoechst 33342 (Beyotime, 100×) in DMEM for 30 min. They were then washed with DMEM to remove the unbound probes. For the competitive assay, cells were pretreated with sildenafil (25 μM) and dipyridamole (25 μM)for 12h respectively and then washed with the above procedure. For siRNA-transfected assay, the cells were transfected with siRNA at concentration of 100 nM with DMEM with 10% FBS without penicillin and streptomycin for 48 h in advance. Fluorescence imaging was then carried out by confocal fluorescence microscope (FV3000,

Olympus). The Fluorescent images were analyzed by the imageJ software and the data were statistical analyzed using GraphPad Prism 8.

4.8 Cell immunofluorescence imaging

LX-2 cells were plated into in a confocal dish at 1×10^5/ per dish in 1 mL medium and allowed to adhere for 8–12 h. Then the cells were fixed at RT for 30min with PFA (Periodate-Lysine-Paraformaldehyde fixative) and washed with PBS (1×, pH 7.4) three times carefully. Then, the fixed cells were treated with Triton X-100 (0.5%, v/v) in PBS (1×, pH 7.4) for another 15 min at RT and washed with PBS (1×, pH 7.4, 2 mL) three times. Afterwards, the cells were blocked with 10% goat serum solution in PBS (1×, pH 7.4) at room temperature for 1 h and washed with PBS (1×, pH 7.4) three times carefully. Subsequently, PDE5A primary antibody (1:500 diluted, Santa Cruz) in goat serum solution (10% in PBS, 300 µL per dish) and PCO2003 (10 µM) was added into dishes and incubated at 4 °C overnight. The cells were washed with PBS (1×, pH 7.4) three times and incubated with goat anti-mouse lgG H&L (Alexa Fluor ® 647(1:1000 diluted, abcam, ab150115) in goat serum solution (10% v/v in PBS, 300 µL per dish) at RT for 1-2 h successively. Finally, the cells were washed with PBS (1×, pH 7.4) three times and stained with DAPI (Solarbio, 1×) at RT for 10-15 min to complete the pretreatment procedure of samples. After DAPI was washed out, the fluorescent images were captured by confocal fluorescence microscope (FV3000, Olympus). The Fluorescent images were also analyzed by the imageJ software. The data were statistical analyzed using GraphPad Prism 8.0.

4.9 Cell transiently transfected with PDE5A siRNA

PDE5A siRNA as well as the transfection reagent used in this experiment was purchased from Guangzhou Ribo Bio Co., Ltd. LX-2 cells were planted into a 6-well cell culture plate at 1×10^5 cell/well. After incubating for 12 h at 37 °C under 5% CO_2 in an incubator, the cells were transfected with siRNA at concentration of 100 nM with DMEM medium with 10% FBS without penicillin and streptomycin. After incubating for 48 h, the relative levels of PDE5A mRNA were determined by qPCR method. Meanwhile, the cells were co-stained with **PCO2003** (10 µM) and Hoechst 33342 for 30 min and the fluorescent images were captured as the aforementioned method.

4.10 Real-time quantitative RT-PCR assays

To determine the efficiency of siRNA transfection, real-time quantitative RT-PCR assay was performed. The primer for PDE5A were designed according to the literature[7] and customized by Shanghai Sangon Biotech Co., Ltd. The primer sequences were as follows:(forward)5’-ATCAGGAAACGGTGGGACATTTAC-3’ and (reverse) 5’ -CTTGTTCTCAGCAGTGAAGTCTC -3’. The primer for β-actin were purchased from

Shanghai Sangon Biotech Co., Ltd. Total mRNA was extracted by RNAiso Plus (9109, Takara) from cells transfected with PDE5A siRNA, and cDNA was produced using the GoScript™ Reverse Transcription System. QRT-PCR analysis was performed on a Roche Light Cycler 480 for real-time PCR. Each reaction was run in triplicate and repeated in at least three independent experiments. Relative expression values for each mRNA were obtained by normalizing them to β-actin expression, and differences between samples were calculated using the $2^{(-\Delta\Delta_{C(T)})}$ method.

4.11 Western Blot assays

The differences of PDE5 expression in various cells were evaluated by western blot assay. LX-2 cells, HeLa cells and A549 cells were planted into a 6-well cell culture plate at 2×10^5/well. Cultured cells were lysised by IP Cell lysis buffer containing protease and phosphatase inhibitors until the cell density reached 90%. The protein concentration was measured by BCA (Thermo Scientific, USA), and samples containing equal amounts of protein (20 µg) were electrophoresed on SDS–polyacrylamide gels. Proteins were then transferred to polyvinylidene fluoride membranes (Millipore, USA). Next, the membranes were blocked with 5% nonfat-dried milk in Tris-buffered saline containing 0.1% Tween-20 (TBST) at room temperature for 1 h, and the membranes were incubated overnight at 4°C with primary antibodies diluted with QuickBlock™ primary antibody dilution buffer(Beyotime).The primary antibodies was as follows: PDE5 antibody (1： 1000 diluted, Santa Cruz) and anti-GAPDH antibody (1:2000 diluted, Santa cruz). The Goat Anti-Mouse lgG secondary antibodies were used at a dilution of 1:3000 at room temperature for 1.5 h, and the detections were performed by using an electrochemiluminescence (ECL) system. The images were analyzed using ImageJ software to obtain the gray value for each band. Then, the relative gray value of PDE5 (gray value of PDE5 /gray value of GAPDH) was calculated. The data were statistical analyzed using GraphPad Prism 8.0.

4.12 Tissue sections immunofluorescence imaging

Tissue section samples were dewaxed and pretreated with the heated citric acid solution for antigen retrieval. Then the samples were blocked with 10% (v/v) goat serum solution in PBS buffer (1×, pH 7.4) for 1 h and incubated with probe **PCO2003** (10 µM), PDE5A primary antibody (1:1000 diluted, Santa Cruz) in 10% (v/v) goat serum solution in PBS at 4 °C overnight and goat anti-mouse lgG H&L (Alexa Fluor ® 647(1:2000 diluted, abcam, ab150115) at RT for 1h successively. Then, the fluorescent images of lung tissue sections were obtained. Images were analyzed by imageJ software.

5. Appendix

^{1}H-NMR spectrum of **1**.

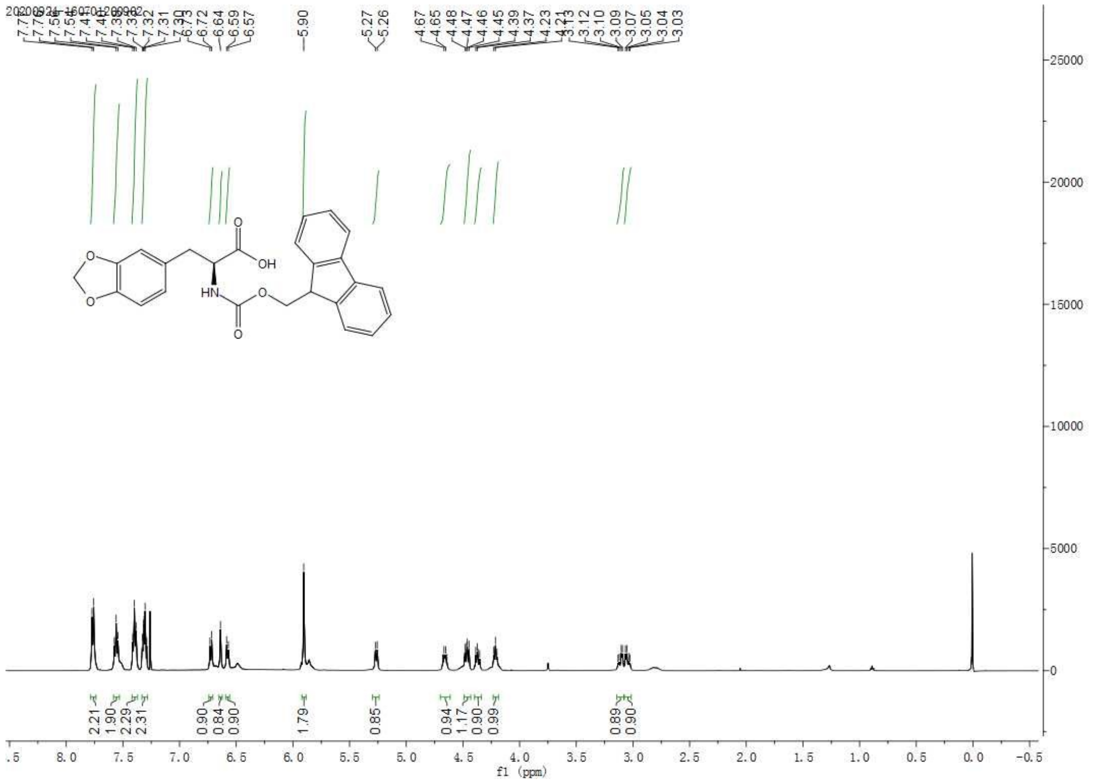

^{1}H-NMR spectrum of **2**.

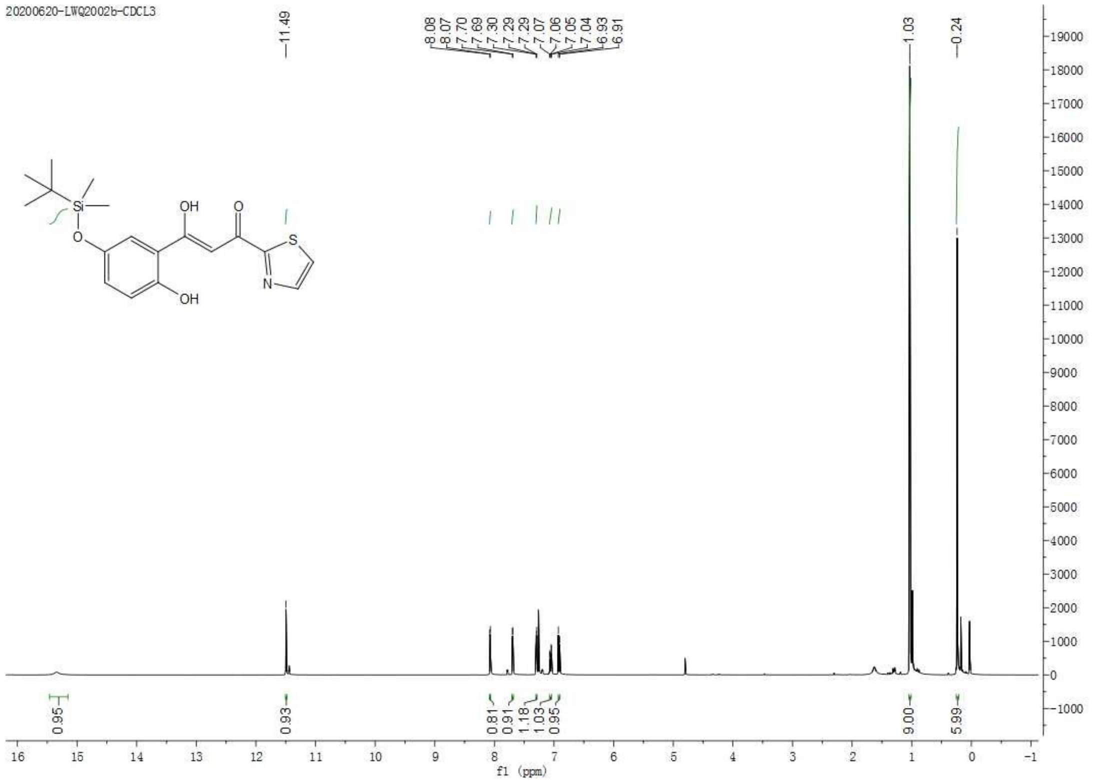

^{1}H-NMR spectrum of **3**.

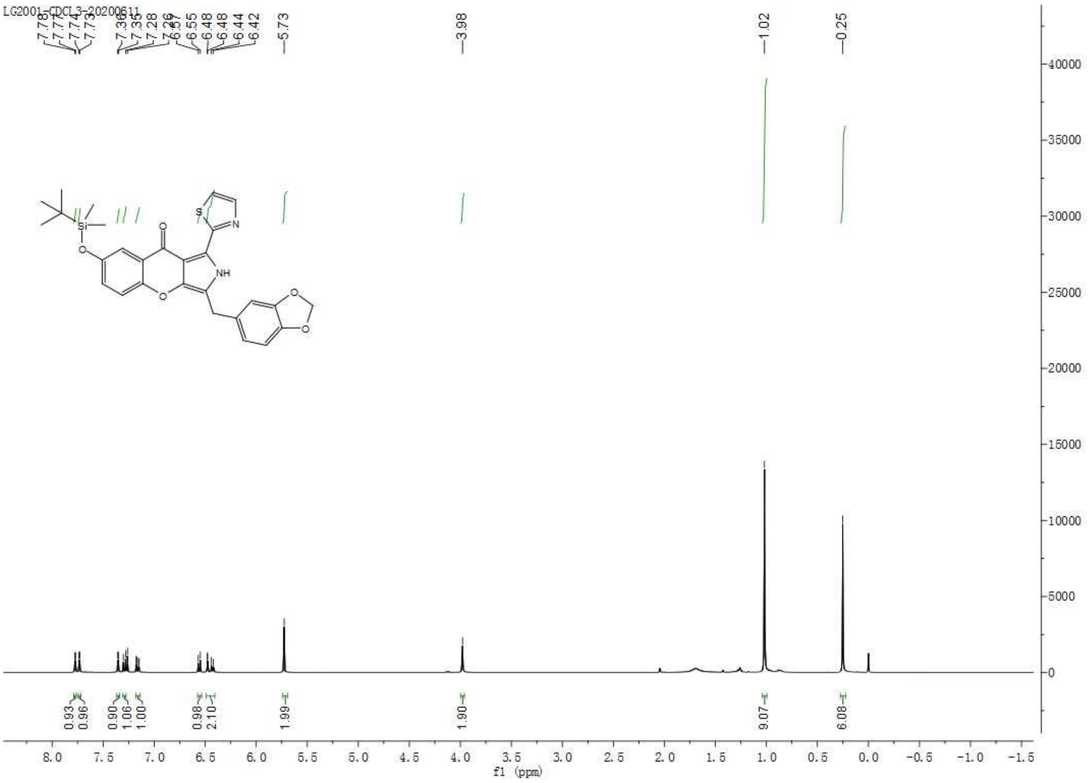

^{1}H-NMR spectrum of **PCO2001**.

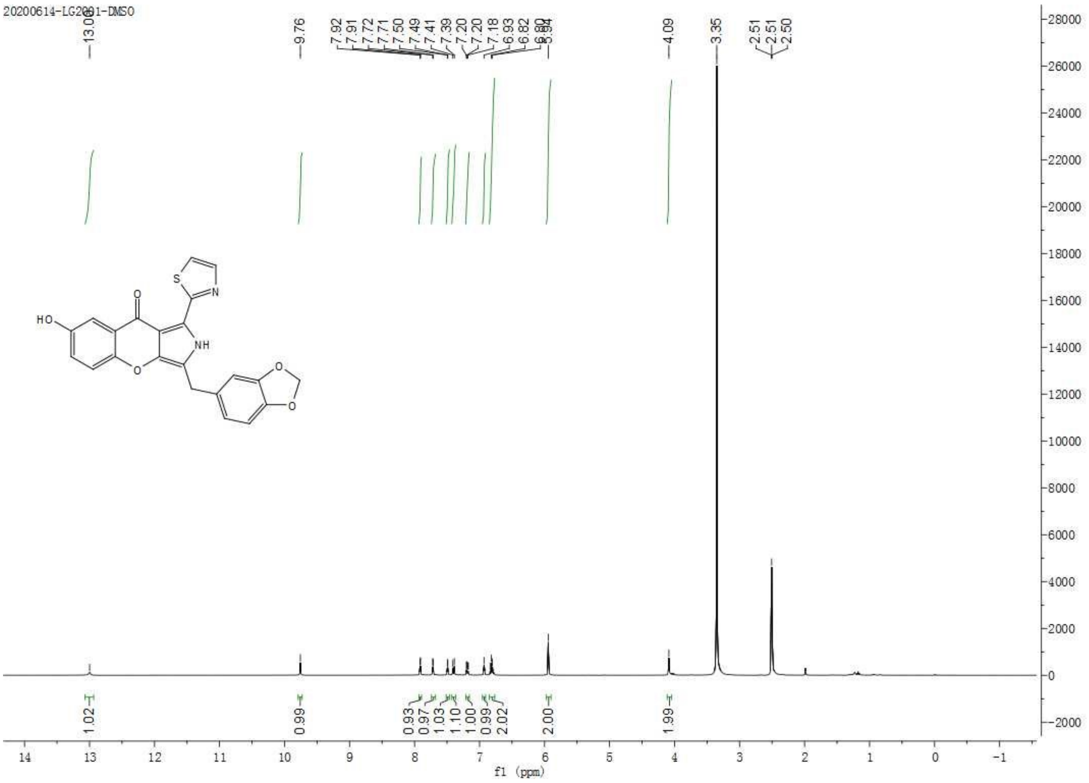

^{13}C-NMR spectrum of **PCO2001**.

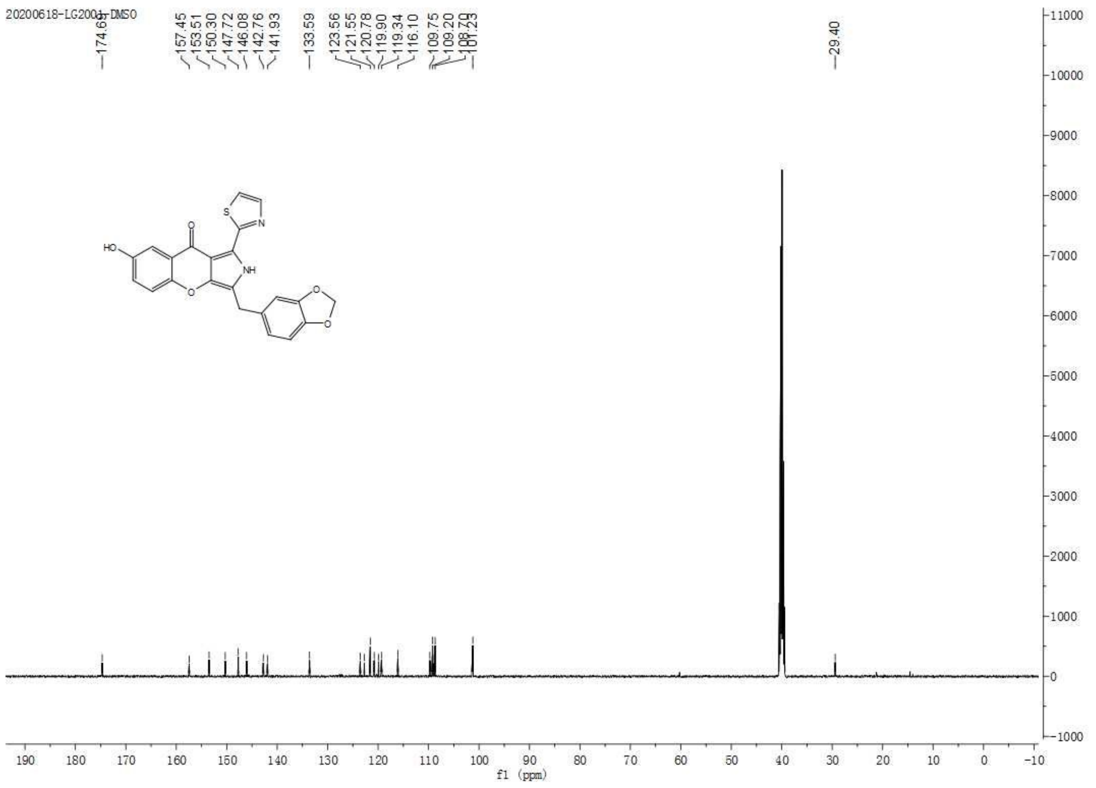

HRMS spectrum of **PCO2001**.

Elmt	Val.	Min	Max	Elmt	Val.	Min	Max	Elmt	Val.	Min	Max	Elmt	Val.	Min	Max	Use Adduct
H	1	14	14	F	1	0	0	Cl	1	0	0	I	3	0	0	K
C	4	22	22	Si	4	0	0	Se	2	0	0	Os	2	0	0	H
N	3	2	2	P	3	0	0	Br	1	0	0	Ir	3	0	0	Na
O	2	5	5	S	2	1	1	Pd	2	0	0					NH4

Error Margin (ppm): 100
HC Ratio: unlimited
Max Isotopes: all
MSn Iso RI (%): 75.00

DBE Range: -100.0 - 100.0
Apply N Rule: yes
Isotope RI (%): 1.00
MSn Logic Mode: OR

Electron Ions: both
Use MSn Info: no
Isotope Res: 10000
Max Results: 50

Event#: 1 MS(E+) Ret. Time : 1.000 Scan# : 151

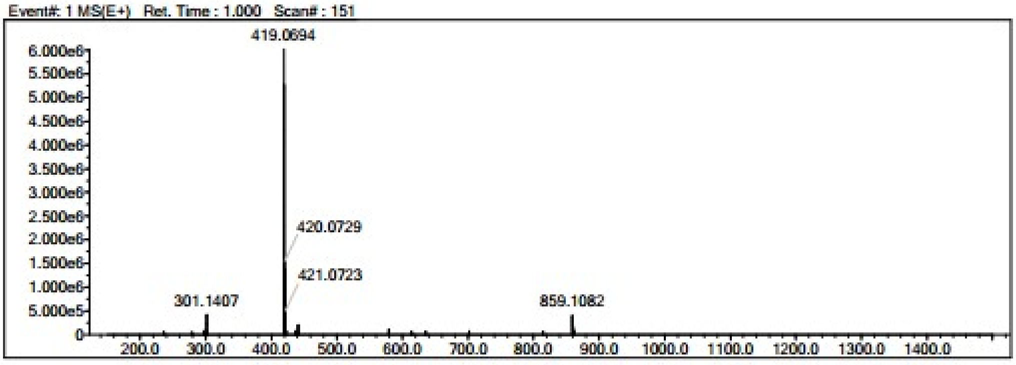

Measured region for 419.0694 m/z

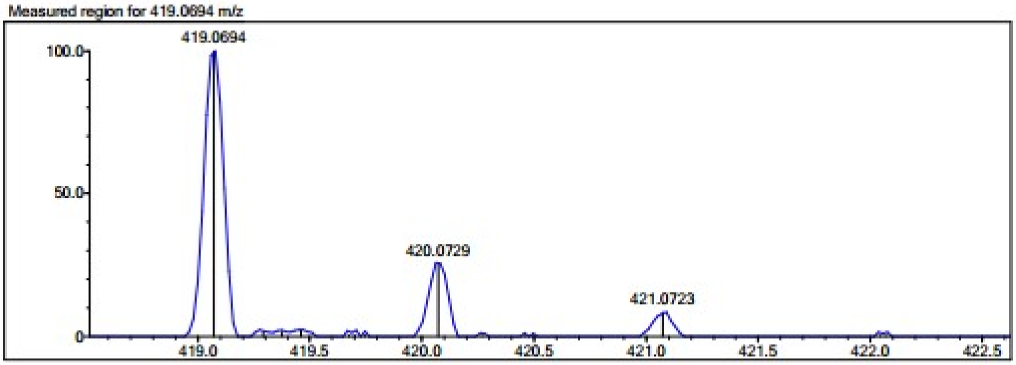

C22 H14 N2 O5 S [M+H]+ : Predicted region for 419.0696 m/z

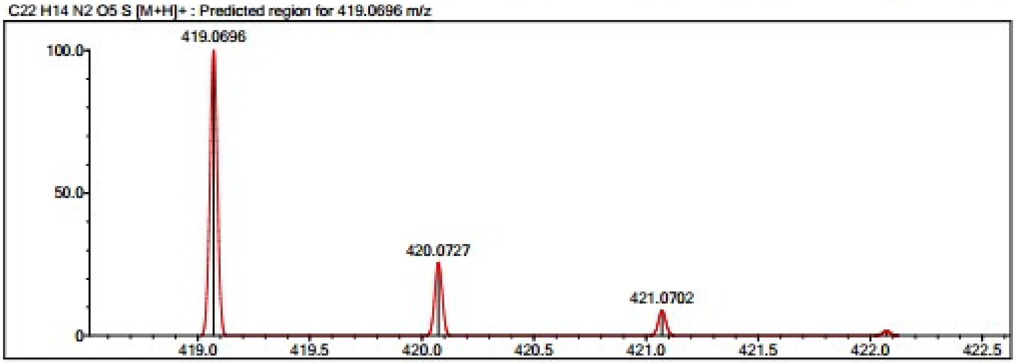

Rank	Score	Formula (M)	Ion	Meas. m/z	Pred. m/z	Df. (mDa)	Df. (ppm)	Iso	DBE
1	90.82	C22 H14 N2 O5 S	[M+H]+	419.0694	419.0696	-0.2	-0.48	90.82	17.0

^{1}H-NMR spectrum of **PCO2002**.

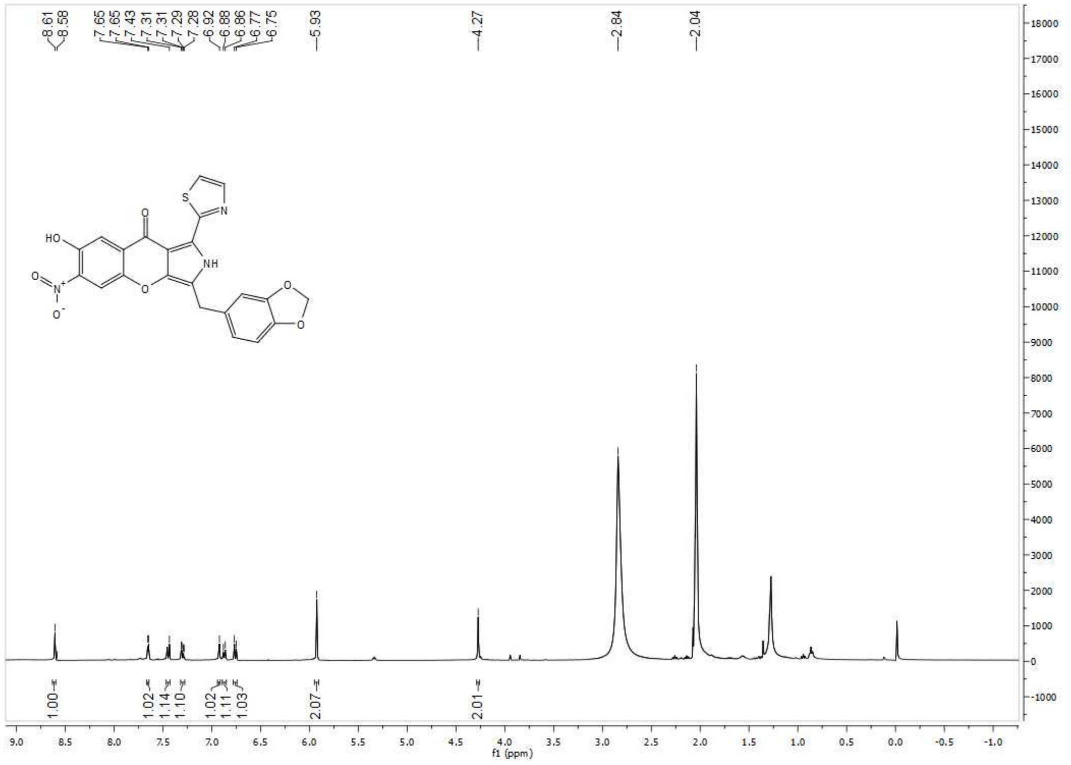

^{13}C-NMR spectrum of **PCO2002**.

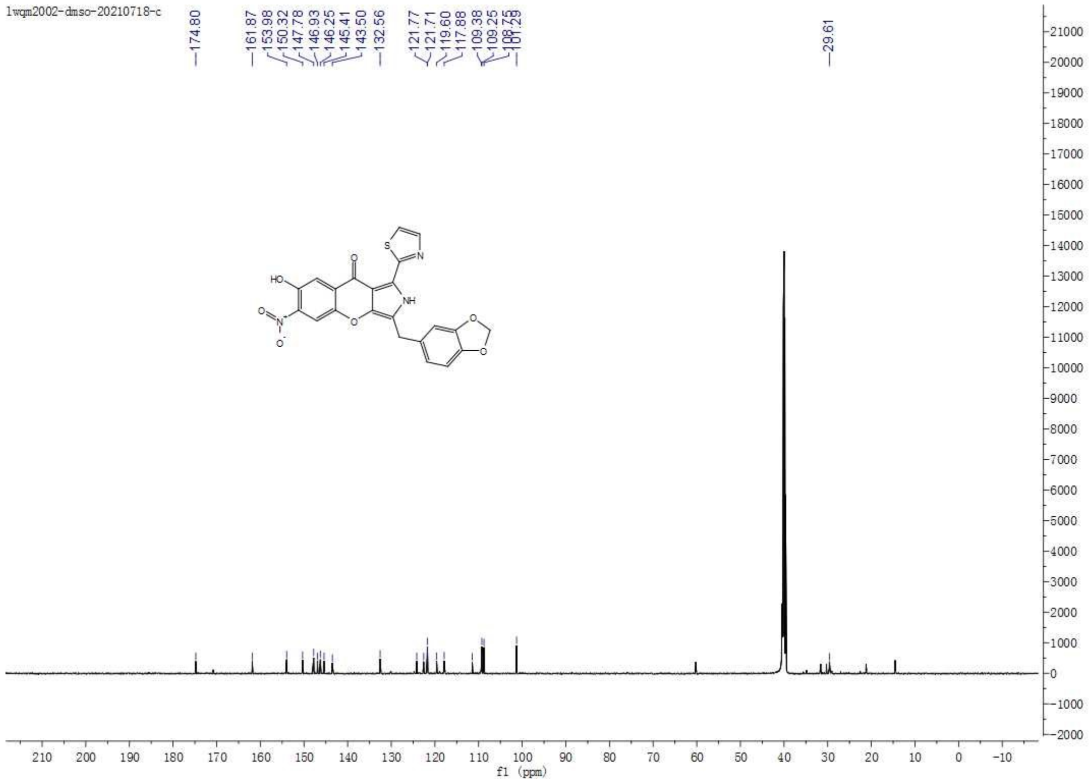

HRMS spectrum of **PCO2002**.

Elmt	Val.	Min	Max	Elmt	Val.	Min	Max	Elmt	Val.	Min	Max	Use Adduct
H	1	0	13	O	2	7	7	Ni	2	0	0	H
2H	1	0	0	F	1	0	0	Se	2	0	0	Na
B	3	0	0	P	3	0	0	Br	1	0	0	NH4
C	4	22	22	S	2	1	1	Sb	3	0	0	K
N	3	3	3	Cl	1	0	0	Pt	2	0	0	

Error Margin (ppm): 100
HC Ratio: unlimited
Max Isotopes: all
MSn Iso RI (%): 75.00

DBE Range: not fixed
Apply N Rule: no
Isotope RI (%): 1.00
MSn Logic Mode: AND

Electron Ions: both
Use MSn Info: no
Isotope Res: 10000
Max Results: 10

Event#: 1 MS(E+) Ret. Time : 1.640 Scan# : 247

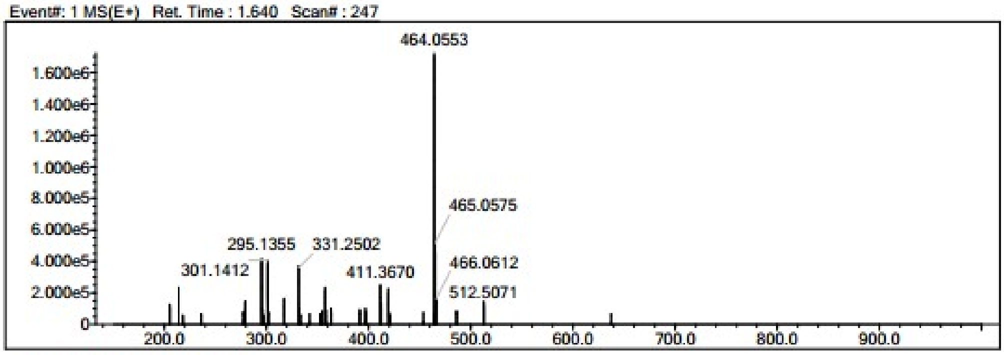

Measured region for 464.0553 m/z

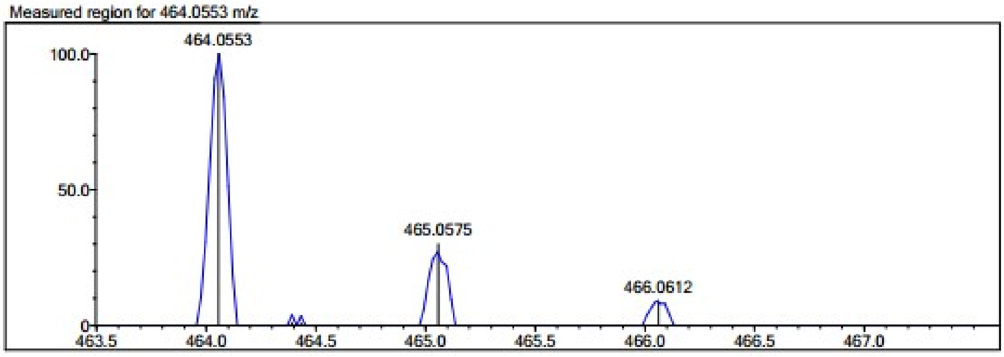

C22 H13 N3 O7 S [M+H]+ : Predicted region for 464.0547 m/z

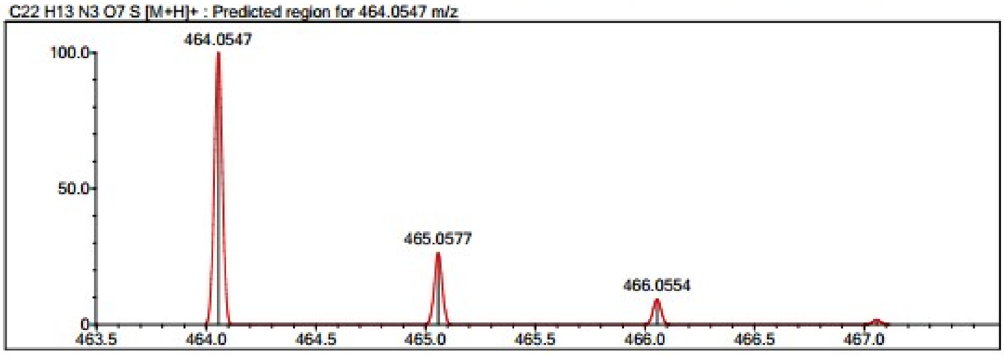

Rank	Score	Formula (M)	Ion	Meas. m/z	Pred. m/z	Df. (mDa)	Df. (ppm)	Iso	DBE
1	95.25	C22 H13 N3 O7 S	[M+H]+	464.0553	464.0547	0.6	1.29	95.95	18.0

^1H NMR spectrum of **5**.

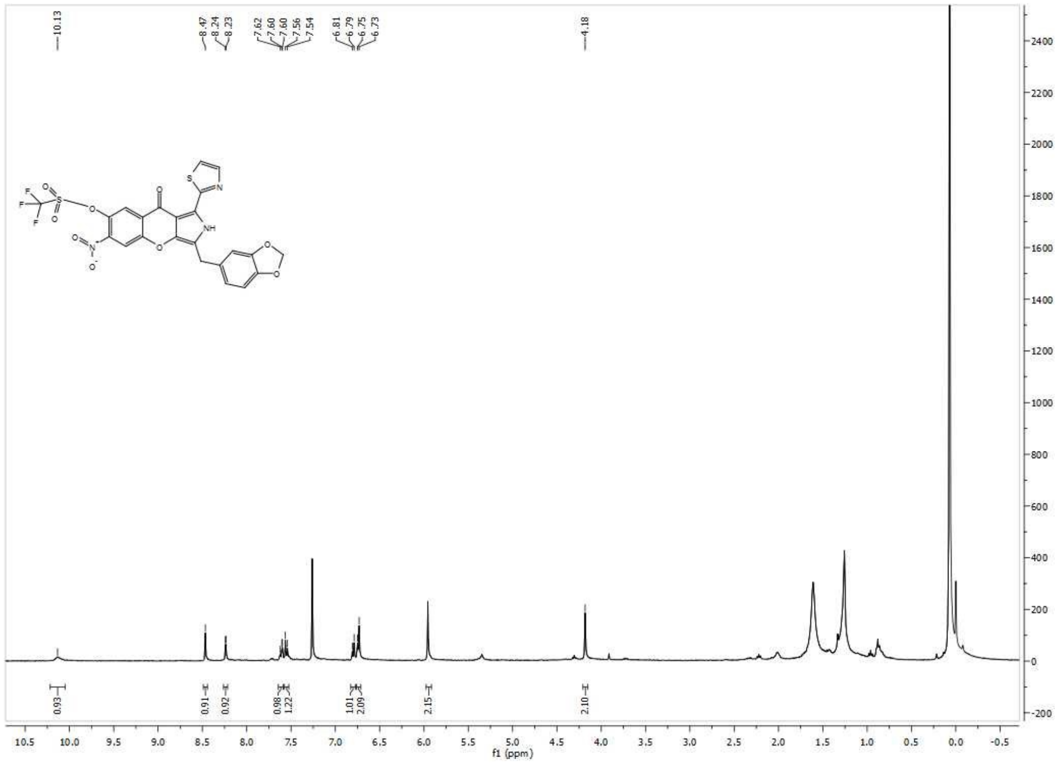

HRMS spectrum of **5**.

Elmt	Val.	Min	Max	Elmt	Val.	Min	Max	Elmt	Val.	Min	Max	Use Adduct
H	1	0	12	F	1	0	3	Cu	2	0	0	H
2H	1	0	0	Si	4	0	0	Se	2	0	0	K
B	3	0	0	P	3	0	0	Br	1	0	0	F
C	4	0	23	S	2	0	2	Sb	3	0	0	Cl
N	3	0	3	Cl	1	0	0	Pt	2	0	0	
O	2	0	9	Ni	2	0	0					

Error Margin (ppm):	20	DBE Range:	not fixed	Electron Ions:	both
HC Ratio:	unlimited	Apply N Rule:	yes	Use MSn Info:	no
Max Isotopes:	all	Isotope RI (%):	1.00	Isotope Res:	10000
MSn Iso RI (%):	75.00	MSn Logic Mode:	AND	Max Results:	500

Event#: 2 MS(E-) Ret. Time : 2.347 Scan# : 354

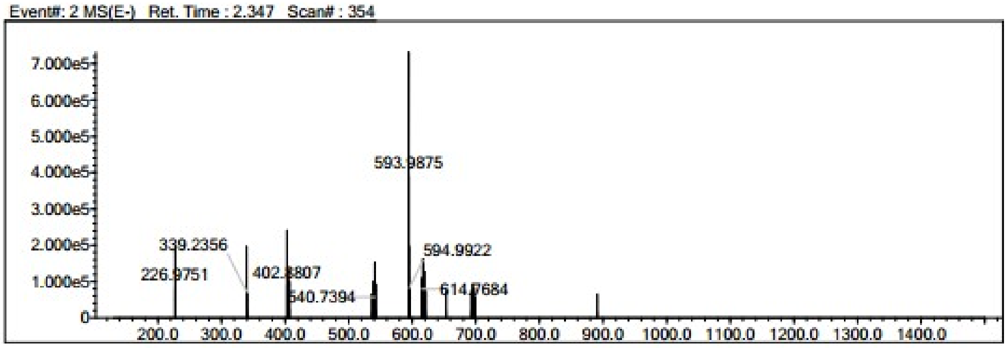

Measured region for 593.9875 m/z

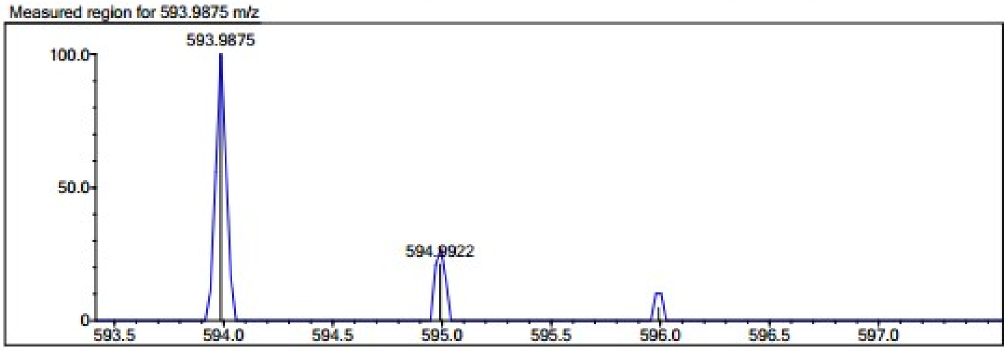

C23 H12 N3 O9 F3 S2 [M-H]- : Predicted region for 593.9894 m/z

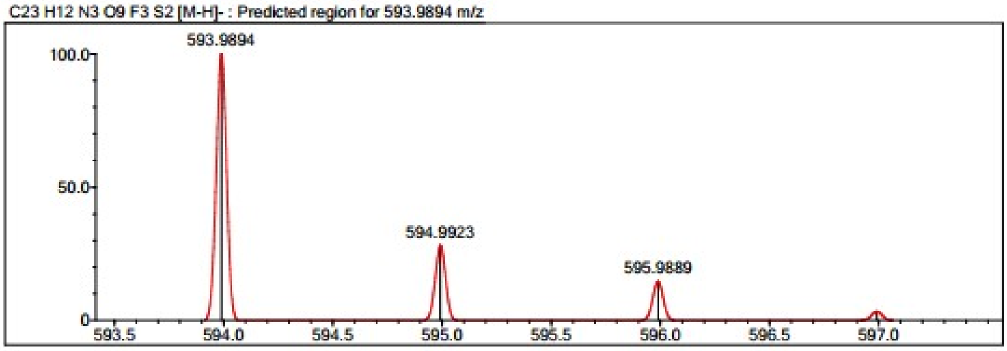

Rank	Score	Formula (M)	Ion	Meas. m/z	Pred. m/z	Df. (mDa)	Df. (ppm)	Iso	DBE
1	75.18	C23 H12 N3 O9 F3 S2	[M-H]-	593.9875	593.9894	-1.9	-3.20	79.55	18.0

^{1}H-NMR spectrum of **PCO2003**.

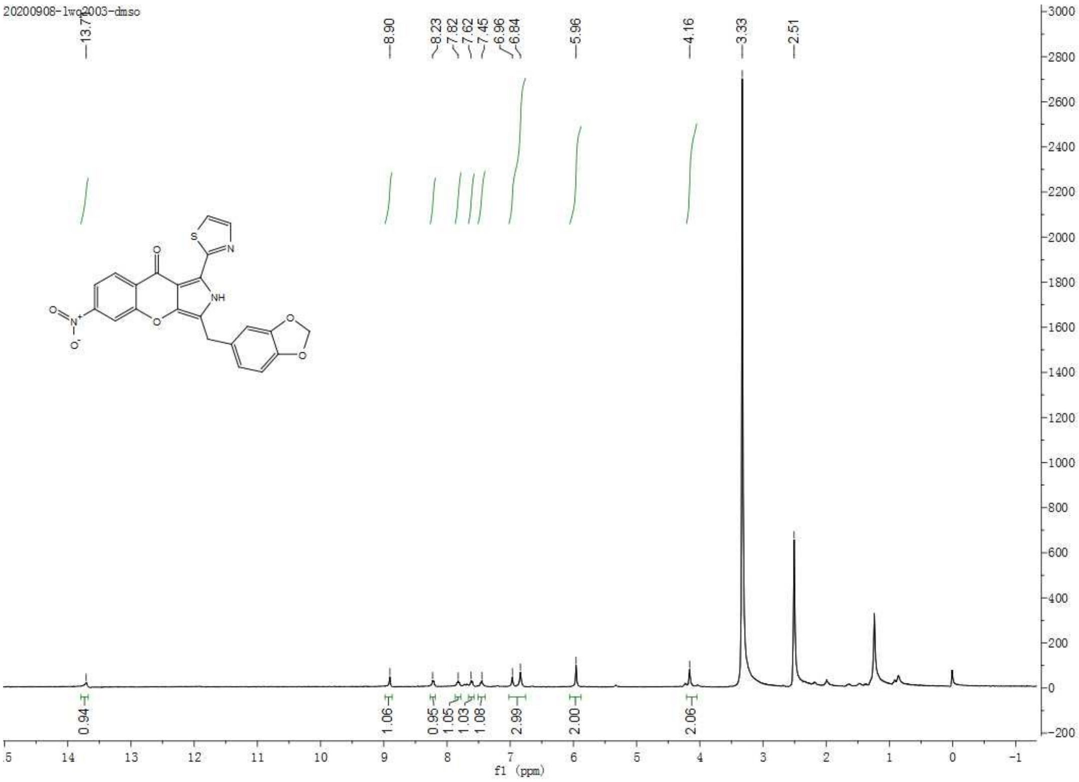

^{13}C-NMR spectrum of **PCO2003**.

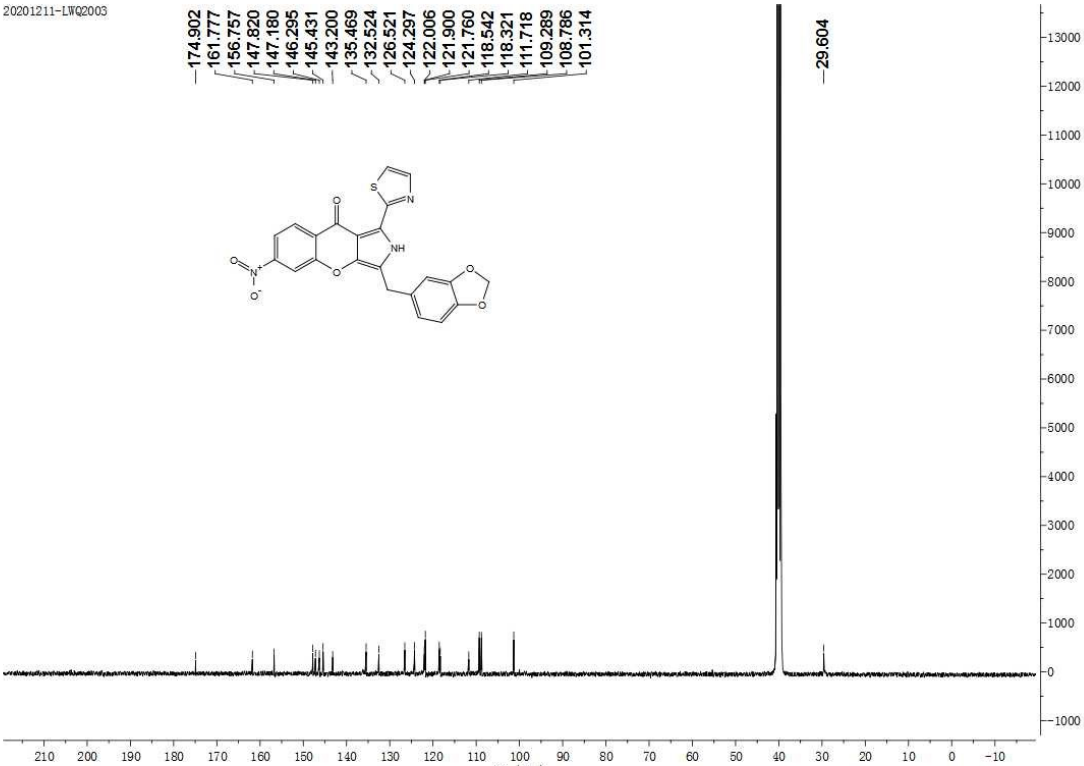

HRMS spectrum of **PCO2003**.

Elmt	Val.	Min	Max	Elmt	Val.	Min	Max	Elmt	Val.	Min	Max	Use Adduct
H	1	0	13	F	1	0	0	Cu	2	0	0	H
2H	1	0	0	Si	4	0	0	Se	2	0	0	Na
B	3	0	0	P	3	0	0	Br	1	0	0	NH4
C	4	0	22	S	2	0	1	Sb	3	0	0	
N	3	0	3	Cl	1	0	0	Pt	2	0	0	
O	2	0	6	Ni	2	0	0					

Error Margin (ppm): 20
HC Ratio: unlimited
Max Isotopes: all
MSn Iso RI (%): 75.00

DBE Range: not fixed
Apply N Rule: yes
Isotope RI (%): 1.00
MSn Logic Mode: AND

Electron Ions: both
Use MSn Info: no
Isotope Res: 10000
Max Results: 500

Event#: 1 MS(E+) Ret. Time : 2.573 Scan# : 387

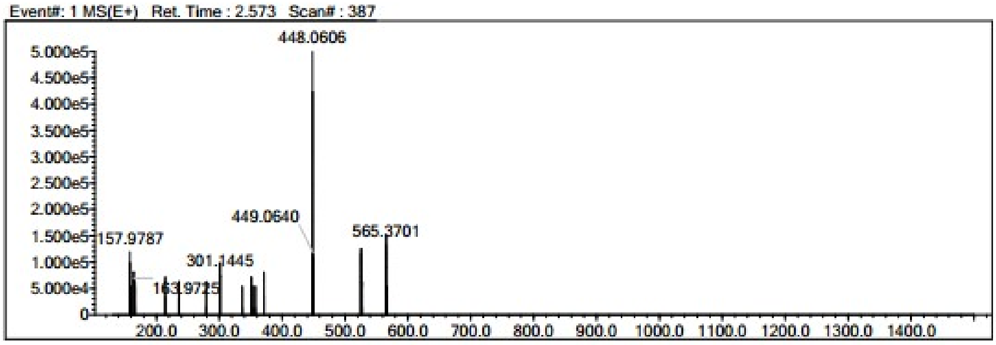

Measured region for 448.0606 m/z

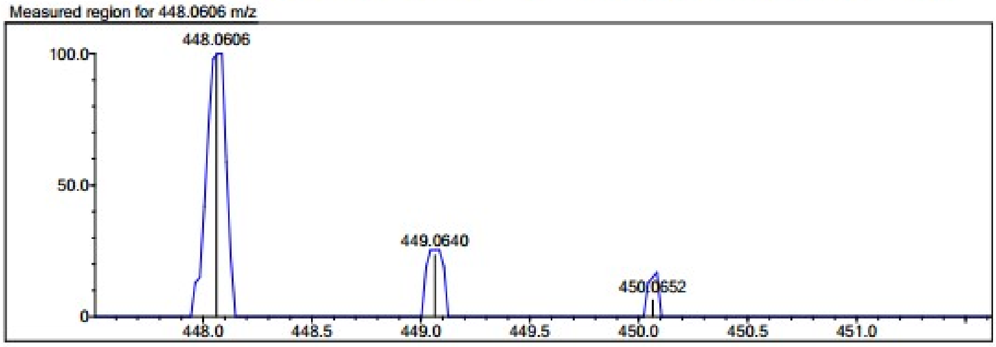

C22 H13 N3 O6 S [M+H]+ : Predicted region for 448.0598 m/z

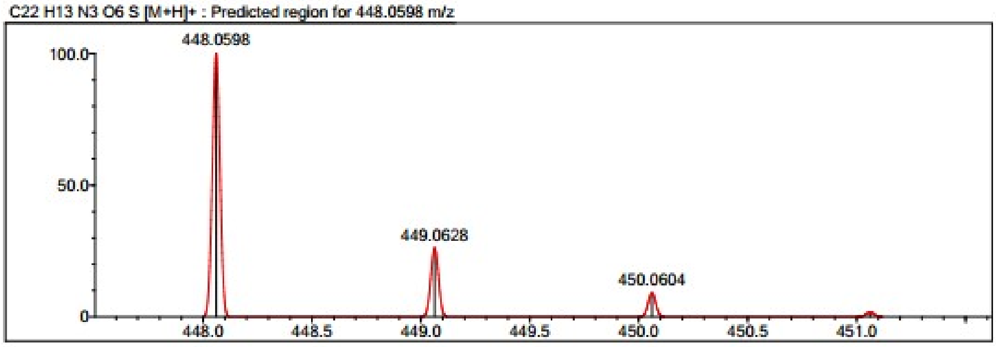

Rank	Score	Formula (M)	Ion	Meas. m/z	Pred. m/z	Df. (mDa)	Df. (ppm)	Iso	DBE
1	59.55	C22 H13 N3 O6 S	[M+H]+	448.0606	448.0598	0.8	1.79	60.75	18.0

The raw data of the Western Blot assays for PDE5A expression in different cells

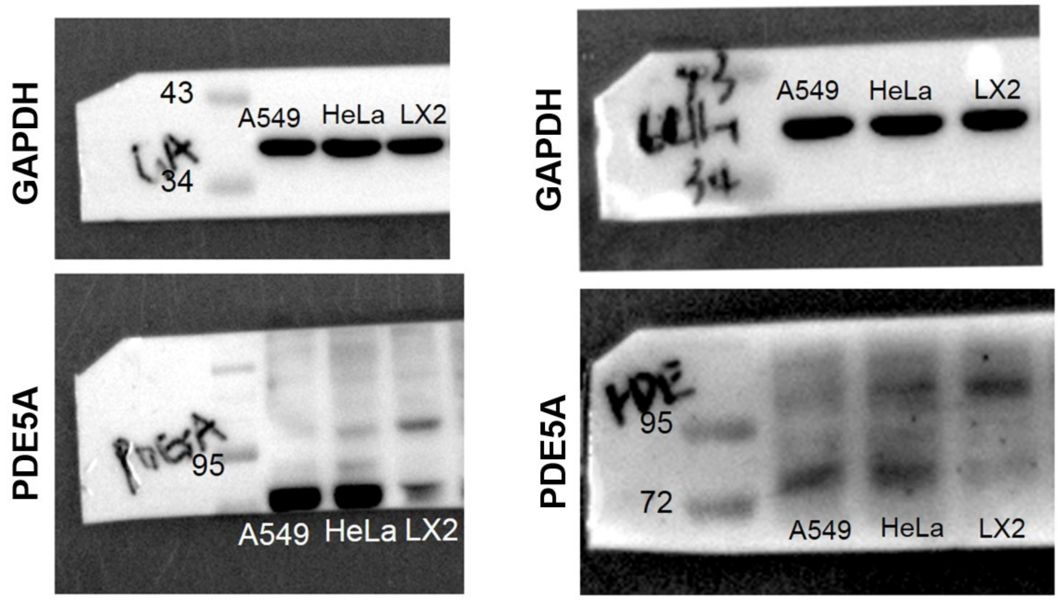

6. References

1. D. Wu, Y. Huang, Y. Chen, Y.-Y. Huang, H. Geng, T. Zhang, C. Zhang, Z. Li, L. Guo, J. Chen and H.-B. Luo, *J. Med. Chem.*, 2018, **61**, 8468-8473.
2. R. Aggarwal, R. G. F. Giles, I. R. Green, F. J. Oosthuizen and C. P. Taylor, *Org. Biomol. Chem.*, 2005, **3**, 263-273.
3. P. Zhou, Y. Liu, L. Zhou, K. Zhu, K. Feng, H. Zhang, Y. Liang, H. Jiang, C. Luo, M. Liu and Y. Wang, *J. Med. Chem.*, 2016, **59**, 10329-10334.
4. A. Patel, F. Liebner, T. Netscher, K. Mereiter and T. Rosenau, *J. Org. Chem.*, 2007, **72**, 6504-6512.
5. Z. Yu, Y. Li, J. Shi, B. Ma, L. Liu and J. Zhang, *Angew. Chem. Int. Edit.*, 2016, **55**, 14807-14811.
6. D. Wu, T. Zhang, Y. Chen, Y. Huang, H. Geng, Y. Yu, C. Zhang, Z. Lai, Y. Wu, X. Guo, J. Chen and H.-B. Luo, *J. Med. Chem.*, 2017, **60**, 6622-6637.
7. V. Lakics, E. H. Karran and F. G. Boess, *Neuropharmacology*, 2010, **59**, 367-374.
